# Assessment of the consistency between YouTube videos and scientific evidence on paediatric myopia control

**DOI:** 10.1371/journal.pone.0357587

**Published:** 2026-09-23

**Authors:** Luis Pérez-Mañá, Carolina Ortiz, Bernat Sunyer-Grau, Silvia Alonso, Marc Argilés

**Affiliations:** 1 Department of Optics and Optometry, Universitat Politècnica de Catalunya - BarcelonaTech (UPC), Terrassa, Spain; 2 Centre for Sensors, Instruments and Systems Development (CD-6), Universitat Politècnica de Catalunya - BarcelonaTech (UPC), Terrassa, Spain; 3 Department of Optics, Clinical and Laboratory Applications of Research in Optometry, University of Granada, Granada, Spain; The Ohio State University, UNITED STATES OF AMERICA

## Abstract

**Background:**

Myopia is a public health concern, and its prevalence is estimated to increase in the near future. Multiple myopia control strategies have been developed to slow myopia progression and reduce potential ocular alterations. Public interest in myopia and its management has increased, with parents seeking information on myopia from available sources, such as the internet. The goal of this study was to systematically evaluate the quality, reliability, and educational value of YouTube videos for myopia control in children and their alignment with current scientific evidence and clinical guidelines.

**Methods:**

A comprehensive search was conducted on YouTube in February 2025 using four primary keywords related to myopia control in children, both in English and Spanish. The first 50 videos for each keyword were screened; of the resulting 400 videos, 235 met the inclusion criteria. Three optometrists independently assessed the videos via the modified DISCERN (mDISCERN), the Journal of the American Medical Association (JAMA) benchmarks, and the Global Quality Score (GQS). Interobserver reliability was evaluated using two-way mixed-effects intraclass correlation coefficients (ICC) with absolute agreement. Quantitative engagement metrics (views, likes, dislikes, the view ratio, and the video power index (VPI) were also recorded. Non-parametric tests were used for subgroup comparisons by language, topic, source, and presenter gender, with Bonferroni adjustments applied for multiple comparisons.

**Results:**

Among the 235 videos analysed, 52.8% were in English, and 47.2% were in Spanish. Overall video quality and reliability were highly variable. Interobserver reliability across the entire dataset was good for GQS (ICC = 0.832) and mDISCERN (ICC = 0.750), but poor for JAMA (ICC = 0.447). Good interevaluator agreement was maintained across all tools for Spanish videos (ICC range: 0.701–0.803), whereas English videos showed lower agreement for mDISCERN (ICC = 0.768) and JAMA (ICC = 0.536), but excellent agreement for GQS (ICC = 0.849). Spanish-language videos scored significantly higher on mDISCERN (P = 0.026), while JAMA and GQS scores showed no significant differences between languages after adjusting for multiple comparisons. Videos from universities or professional organisations were of higher quality and reliability but constituted a minority of the sample. The presenter’s gender did not impact quality or engagement. English-language videos had higher view ratios and VPI values, indicating greater visibility, although not necessarily higher scientific quality.

**Conclusions:**

YouTube provides a wide range of content on pediatric myopia control, but its overall quality, realiability and scientific alignment are inconsistent. The use of standardized evaluation tools reveals significant differences in subjective assessment depending on the scoring instrument and video language. The increased participation of healthcare professionals in creating evidence-based videos is needed to increase public health literacy.

## Introduction

Myopia, or nearsightedness, is a growing public health concern worldwide, especially among children and adolescents. The prevalence of myopia has increased dramatically in recent decades, and projections indicate that nearly half of the global population may be affected by myopia by 2050, with up to 10% experiencing high myopia [1,2]. This alarming trend is largely attributed to environmental and behavioural factors, such as increased near work and reduced time spent outdoors [3]. Early-onset myopia is associated with a greater risk of developing high myopia and sight-threatening complications later in life, including retinal detachment, myopic maculopathy, glaucoma, and choroidal neovascularisation. Therefore, identifying and implementing effective strategies for myopia control during childhood is particularly important [4,5].

Myopia control has been a topic of great interest among researchers for more than two decades [6]. Initially, parental concern about myopia progression in their children was limited [7]. However, in recent years, there has been a significant increase in internet searches related to myopia, particularly on video platforms such as YouTube, reflecting greater awareness and concern among parents [8].

YouTube, as one of the most popular video-sharing platforms, has far-reaching divulgative power and plays a pivotal role in shaping public perceptions and health behaviours. However, the reliability and scientific accuracy of the information it provides are often uncertain, with previous research highlighting considerable variability and frequent deviations from evidence-based recommendations [9–12]. In ophthalmology, the educational quality of YouTube content has only recently begun to receive attention, with preliminary findings indicating significant inconsistency between online materials and evidence-based recommendations [13,14]. Given the impact that online information can have on health-related decisions, particularly among concerned parents, it is essential to assess the quality and accuracy of publicly available content and its adherence to established clinical guidelines.

Therefore, this study aimed to systematically review and analyse the content of YouTube videos focused on myopia control in children, assessing their quality, reliability, and educational value. By comparing the information presented in these videos with current scientific evidence and established clinical guidelines, [15,16] we sought to determine the extent to which the content available to parents aligns with evidence-based recommendations. To the best of our knowledge, this is the first targeted evaluation of the consistency between scientific research and publicly accessible information on YouTube regarding childhood myopia control.

## Methods

### Search strategy

This descriptive, cross-sectional, record-based study was conducted on February 11,2025. As no personal data were collected or processed, ethical approval and informed consent to participate were not required. First, Google Trends, a publicly accessible platform that tracks the frequency of specific search queries in real time, was employed to identify pertinent keywords. The phrase “Myopia control in children” served as the primary query, and the search parameters were set as follows: global scope, the preceding five years, all categories, and YouTube as the search source. The most relevant search terms were selected from these results.

To further refine the keyword selection, Semrush, a dedicated tool for Search Engine Optimisation (SEO), was used. This application enables the evaluation of related keywords, focusing on those with the highest search volumes. The proposed keywords were discussed among five members of the research team, all of whom were optometrists. Four keywords were ultimately selected for use in the studies in English and Spanish versions: Myopia control in children (“Control de miopía en niños”), Contact lenses for myopia control (“Lentes de contacto para control de miopía”), Spectacle lenses for myopia control (“Gafas para control de miopía”), Atropine eye drops for myopia (“Atropina en gotas para control de miopía”). English and Spanish were selected because they are among the four most widely spoken languages worldwide.[17]

Searches were performed in private browsing mode, first in English and then in Spanish, using the default search configurations for the United States and Spain, respectively. The search history, cookies, and cached data were deleted beforehand. To minimise potential biases and ensure reproducibility, all YouTube searches (www.youtube.com) were conducted without logging into any account, maintaining the platform’s default sorting method (by relevance). The initial screening for both languages was carried out by a single researcher, who reviewed the first 50 videos for each keyword, as studies have shown that 95% of users rarely go beyond the initial search results.[18]

A total of 400 videos were compiled into a dedicated YouTube library for subsequent detailed evaluation. Videos were excluded if they were not in English (for English selection) or not in Spanish (for Spanish selection). Videos with comments or like/dislike features disabled were also excluded, as these typically have lower engagement and are less likely to appear prominently in the search results. Furthermore, the absence of enabled-like and dislike functionalities prevented proper assessment using one of the main metrics in this study. Additionally, videos that were unrelated to any of the keyword searches or were duplicate entries for each keyword were excluded. After the initial screening, a second researcher reviewed and cleaned the database to remove any remaining ineligible or duplicate entries. A comprehensive list of all the included videos is provided in the appendix (S1 Table). All data collection and analysis methods complied strictly with the Terms of Service and guidelines of YouTube. No automated scraping tools that violate the platform’s policies were used.

### Video assessment

The next stage of the research was conducted by three optometrists (C.O., B.S.-G. and S.A.) with expertise in myopia control working independently and without knowledge of each other’s assessments. All three examiners were from Spain, with Spanish as their native language and C1-level proficiency in English. For all videos meeting the inclusion criteria, a range of characteristics were documented: origin, URL, title, view count, number of likes and dislikes, comment count, time since upload, gender of the main individual featured, view ratio (total views/days since upload), and like ratio (likes × 100/[likes + dislikes]). One such measure was the video power index (VPI), calculated as [view ratio × like ratio]/100, which integrates viewing frequency and the proportion of positive ratings to gauge overall video popularity, as previously described in the literature [19]. To ensure privacy, the identities of the individuals or organisations uploading the videos were not collected.

### Assessment scales

The quality and reliability of the content were assessed with three distinct scoring systems: the modified DISCERN tool (mDISCERN), [20] the Journal of the American Medical Association (JAMA) benchmark criteria, [21] and the Global Quality Score (GQS) [22]. The mDISCERN tool examines five essential aspects: clarity, reliability, impartiality, presentation of additional sources, and certainty of content, with each aspect scored with either 0 or 1, resulting in a total score ranging from 0 to 5 [23]. Higher mDISCERN scores indicate superior quality and reliability, suggesting that videos are trustworthy sources of information for both healthcare professionals and the general public.

The JAMA benchmark, in contrast, evaluates authorship, attribution, disclosure, and currency, with each category scored individually from 0, minimum, to 1, maximum. The final scores range from 0 to 4: a score of 1 reflects inadequate source information, 2–3 indicates partial sufficiency, and 4 denotes fully sufficient source details [21]. The GQS provides a single overall rating of the educational quality of the audiovisual material, using a scale from 1 to 5, where 1 represents poor quality and usefulness and 5 represents the highest educational value [22]. The results for each scoring system are presented in Table 1.

**Table 1:** Summary of the evaluation criteria used: mDISCERN, JAMA, and GQS.

mDISCERN score (range from 0 to 5)	
1. Are the aims clear and achieved?	
2. Are reliable sources of information used? (i.e., publication cited, speaker is board-certified vascular surgeon)	
3. Is the information presented balanced and unbiased?	
4. Are additional sources of information listed for patient reference?	
5. Are areas of uncertainty mentioned?	
**JAMA criteria (range from 0 to 4)**	
Criteria	Description
Authorship	Authors and contributors, their affiliations, and relevant credentials should be provided
Attribution	References and sources for all content should be listed clearly, and all relevant copyright information noted
Disclosure	Website “ownership” should be prominently and fully disclosed, as should any sponsorship, advertising, underwriting, or commercial funding
Currency	Dates that content was posted and updated should be indicated
**Global Quality Score (GQS) (range from 1 to 5)**	
1. Poor quality; very unlikely to be of any use to patients	
2. Poor quality but some information present; of very limited use to patients	
3. Suboptimal flow, some information covered but important topics are missing; somewhat useful to patients	
4. Good quality and flow, most important topics covered; useful to patients.	
5. Excellent quality and flow; highly useful to patients	

Videos were organised into four primary categories for each language, based on the initial search terms: myopia control in children, contact lenses for myopia control, spectacle lenses for myopia control, and atropine eye drops for myopia, in English; and “control de miopía en niños”, “lentes de contacto para control de miopía”, “gafas para control de miopía”, and “atropina en gotas para control de miopía”, in Spanish. Additionally, the videos were classified into the following three subgroups on the basis of the affiliation of the author or publisher: (1) universities or professional organisations, (2) medical or commercial companies, and (3) independent individuals. Each YouTube video was reviewed and classified according to the gender of the main presenter. The classification was based on the following categories: female, male, both male and female as main presenters, animated characters, or undetermined. The “undetermined” category was used when the main presenter did not appear on the screen and gender could not be reliably identified by voice or otherwise,

Video accessibility was evaluated according to certain guidelines set by the Web Accessibility Initiative (WAI) [24] of the Worldwide Web Consortium (W3C), specifically referencing the Web Content Accessibility Guidelines (WCAG) 2.1 [25]. This assessment included checking for features such as subtitles, sign language interpretation, audio descriptions, text transcriptions, detailed content descriptions, and the usability of playback controls (play, pause, rewind). The presence of highlighted key moments to aid navigation was also considered.

Many videos address multiple topics, making categorisation by specific content unfeasible. Therefore, each video was assessed on the basis of how effectively it met its stated objectives, the relevance of its title, and the overall quality of its content.

All the data and scores were recorded independently by the three evaluators, who were masked to each other’s assessments to minimize bias. As this research involved only publicly accessible YouTube videos and did not involve direct contact with patients, approval from an ethics committee was not needed, in line with similar studies.[12,26,27] The average scores from the three reviewers were then subjected to statistical analysis.

### Statistical analysis

Statistical analyses were conducted via IBM SPSS Statistics for Windows, version 28 (IBM Corp., Armonk, NY,USA). Given the ordinal nature of the scores obtained from the assessment scales (mDISCERN, JAMA, and GQS), non-parametric analyses were inherently indicated. Additionally, data distribution was assessed via the Kolmogorov–Smirnov test for the continuous video metrics (including total duration, number of views, likes, dislikes, comments, like ratio, view ratio, and VPI), which confirmed a non-normal distribution (*P* <0.001 for all). Therefore, descriptive statistics are presented as medians with interquartile ranges (25th to 75th percentiles). Interobserver reliability for the evaluation scores was assessed via the intraclass correlation coefficient (ICC), using a two-way random-effects model based on absolute agreement and single measurements [28]. ICC values were interpreted as follows: less than 0.50 indicates poor reliability, between 0.50 and 0.75 indicates moderate reliability, between 0.75 and 0.90 indicates good reliability, and greater than 0.90 indicates excellent reliability [29]. Comparisons between video characteristics based on language, presenter gender, source group, and topic were performed via the Kruskal–Wallis test and the independent-samples Mann–Whitney U test. To protect against Type I error inflation due to multiple pairwise comparisons, the Bonferroni correction was applied to adjust the statistical significance thresholds.

## Results

**Fig 1:**
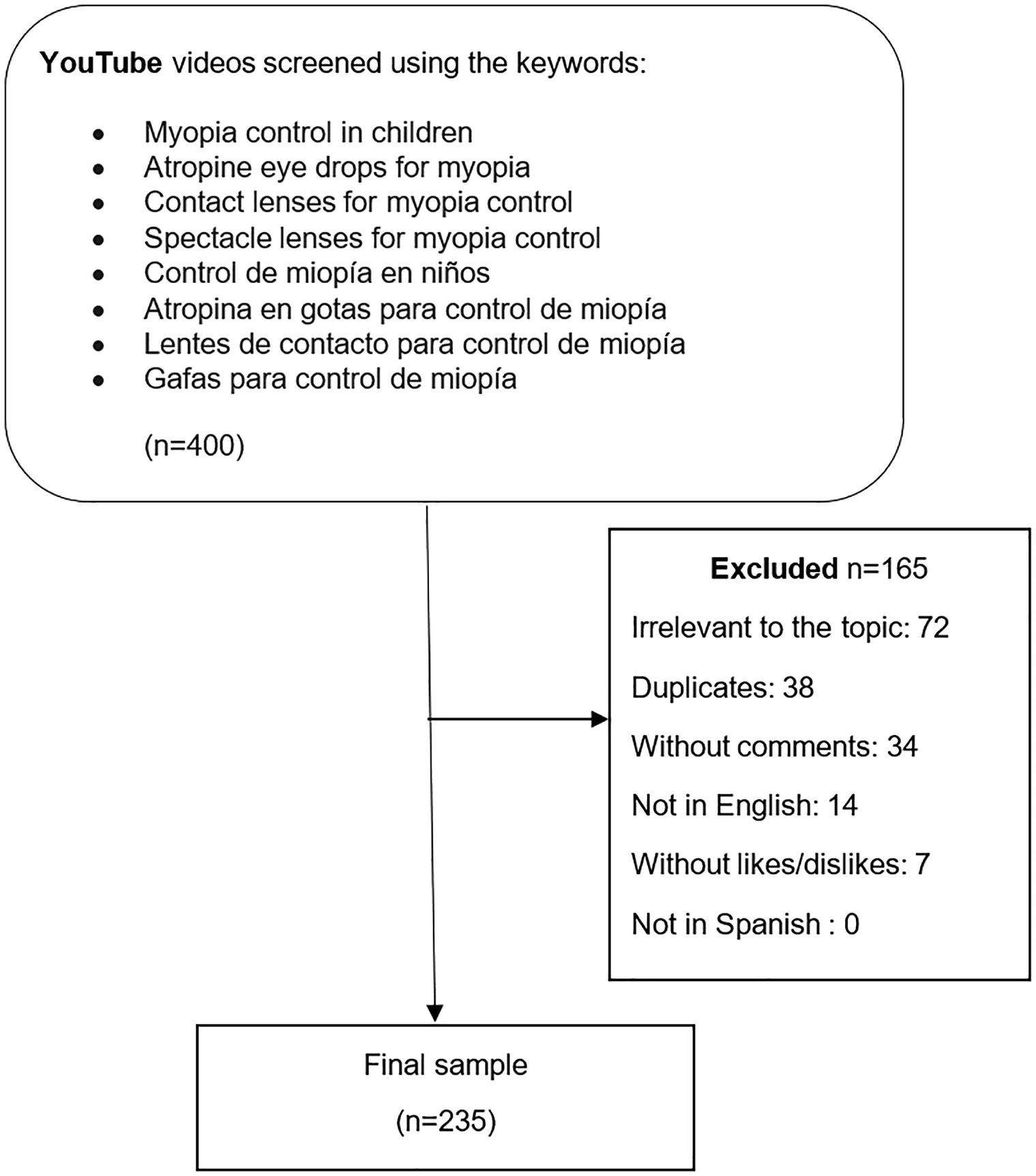
Flowchart of the process of selecting videos from YouTube.

Among the initial 400 videos identified through the search process, 235 met the inclusion criteria and were included in the analysis. Of these, 124 videos (52.8%) were in English, and 111 (47.2%) were in Spanish. Fig 1 illustrates the selection process and shows the number of videos excluded at each stage on the basis of the established criteria.

Among the 235 videos analysed, 222 included an audio-video description (subtitles), providing a synchronised combination of spoken narration and visual content, whereas 13 did not. Additionally, 185 videos featured video transcription; the video’s audio was automatically translated into text, whereas 50 videos lacked this feature. Table 2 summarises the distribution of videos by language, main topics, and key descriptive features such as the presence of audio-video descriptions and transcriptions.

**Table 2:** Summary of included videos by Language, Topic, and Descriptive Characteristics in number and percentage in parentheses (%).

Characteristics	Total (n = 235)	English (n = 124)	Spanish (n = 111)
Language Distribution		124 (52.8%)	111 (47.2%)
Main Topic of the Video			
Myopia control in children	80 (34.0%)	40 (50.0%)	40 (50.0%)
Atropine eye drops	68 (28.9%)	32 (47.1%)	36 (52.9%)
Contact lenses for myopia control	49 (20.9%)	28 (57.1%)	21 (42.9%)
Spectacle lenses for myopia control	38 (16.2%)	24 (63.2%)	14 (36.8%)
Audio-Video Description			
Includes audio-video description (subtitles)	222 (94.5%)	114 (51.3%)	108 (48.7%)
Include description	225 (95.7%)	119 (52.9%)	106 (47.1%)
Include transcription	185 (78.7%)	83 (44.9%)	102 (55.1%)
Include highlighted key moments	66 (28.1%)	39 (59.1%)	27 (40.9%)

When stratified by language, English-language videos demonstrated moderate to good interobserver reliability across all instruments, with ICC values of 0.768 for mDISCERN, 0.536 for JAMA, and 0.849 for GQS. Spanish-language videos showed good reliability for mDISCERN (ICC = 0.701) and GQS (ICC = 0.803), whereas JAMA demonstrated lower reliability (ICC = 0.319). Overall, GQS exhibited the highest reliability in both language groups.These findings are detailed in Table 3.

**Table 3:** Interobserver agreement and reliability were measured by the Intraclass correlation coefficient (ICC) and 95% confidence interval (CIs).

	mDISCERN	JAMA	GQS
**All videos**	0.750 95%CI (0.70–0.79)	0.447 95%CI (0.29–0.57)	0.832 95%CI (0.80–0.86)
**English videos**	0.768 95%CI (0.70–0.83)	0.536 95%CI (0.35–0.67)	0.849 95%CI (0.80–0.89)
**Spanish videos**	0.701 95%CI (0.62–0.77)	0.319 95%CI (0.17–0.46)	0.803 95%CI (0.74–0.85)

### Quality of videos between languages

The results from the three questionnaires (mDISCERN, JAMA, and GQS), along with quality metrics such as likes, dislikes, view ratio, like ratio, and VPI, were analysed separately for English and Spanish-language videos. The Mann‒Whitney test showed no statistically significant differences in mDISCERN (adjusted P = 0.208), JAMA (adjusted P = 0.496), or GQS scores (adjusted P = 1.000) between English- and Spanish-language videos after Bonferroni correction for multiple comparisons. Regarding the video quality metrics, no statistically significant differences were observed between languages in the number of likes (adjusted P = 1.000), number of dislikes (adjusted P = 1.000), like ratio (adjusted P = 1.000), view ratio (adjusted P = 0.328), or VPI (adjusted P = 0.248). Descriptive statistics and adjusted P values are presented in Table 4.

**Table 4:** Descriptive statistics (median and interquartile range) between mDISCERN, JAMA, and GQS, and metrics such as Likes, Dislikes, Like ratio, View ratio, and VPI between English- and Spanish-language videos. The last column shows the the adjusted p-values from the Kruskal‒Wallis test with Bonferroni’s correction.

	English (n = 124)	Spanish (n = 111)	*P* ‒ value
**mDISCERN**	2.33 (1.33,3.67)	2.67 (2.33,3.33)	*P* = 0.208
**JAMA**	1.67 (1.00,2.33)	2.00 (1.67,2.33)	*P* = 0.496
**GQS**	3.00 (1.67,3.67)	3.00 (2.33,3.33)	*P* = 1.000
**Likes**	28.50 (5.75–187.5)	29.00 (5.00,106.00)	*P* = 1.000
**Dislikes**	0.00 (0.00,3.00)	0.00 (0.00,1.00)	*P* = 1.000
**Like ratio**	100.00 (98.33,100.00)	100.00 (99.36,100.00)	*P* = 1.000
**View ratio**	2.91 (0.63,19.48)	1.99 (0.37,8.23)	*P* = 0.328
**VPI**	2.87 (0.61,19.29)	1.97 (0.37,8.21)	*P* = 0.248

For the English videos, according to the mDISCERN criterion, 25 videos (20.2%) scored between 0 and 1, 25 videos (20.2%) scored between 1 and 2, 29 videos (23.4%) scored between 2 and 3, 24 videos (19.4%) scored between 3 and 4, and 21 videos (16.9%) scored between 4 and 5, out of a total of 124 videos. For the JAMA criterion, 32 videos (25.8%) scored between 0 and 1, 49 videos (39.5%) scored between 1 and 2, 34 videos (27.4%) scored between 2 and 3, 9 videos (7.3%) scored between 3 and 4, and none scored between 4 and 5. According to the GQS criterion, 18 videos (14.5%) were rated between 0 and 1, 21 videos (16.9%) were between 1 and 2, 37 videos (29.8%) were between 2 and 3, 23 videos (18.5%) were between 3 and 4, and 25 videos (20.2%) were between 4 and 5.

**Fig 2:**
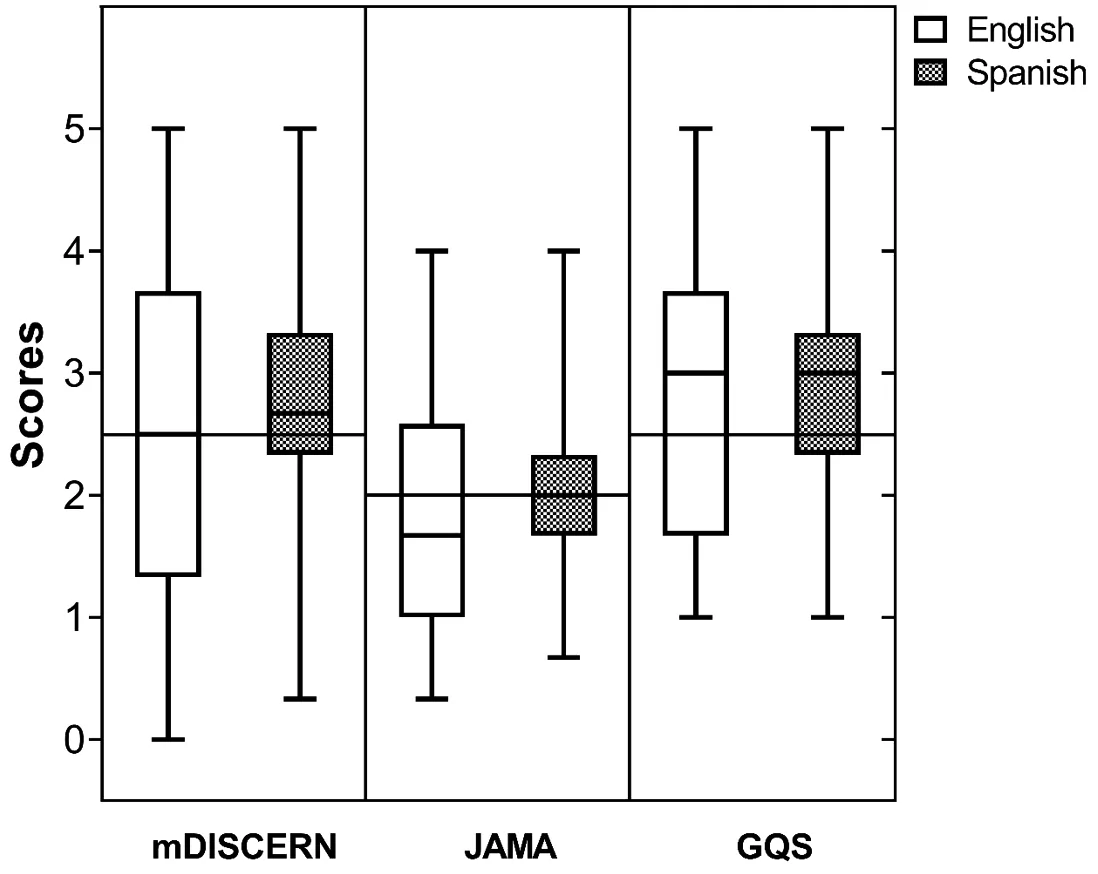
Box plots of the mDISCERN, JAMA, and GQS score results for each language are presented. The results are shown as medians, with minimum and maximum values. The horizontal lines on each plot represent the thresholds that distinguish between good- and poor-quality samples [21].

For the Spanish videos, on the basis of the mDISCERN criterion, 6 videos (5.4%) scored between 0 and 1, 16 videos (14.4%) scored between 1 and 2, 55 videos (49.5%) scored between 2 and 3, 16 videos (14.4%) scored between 3 and 4, and 18 videos (16.2%) scored between 4 and 5. According to the JAMA criterion, 7 videos (6.3%) scored between 0 and 1, 70 videos (63.1%) scored between 1 and 2, 22 videos (19.8%) scored between 2 and 3, 12 videos (10.8%) scored between 3 and 4, and none scored between 4 and 5. For the GQS criterion, 5 videos (4.5%) were rated between 0 and 1, 19 videos (17.1%) were rated between 1 and 2, 58 videos (52.3%) were rated between 2 and 3, 18 videos (16.2%) were rated between 3 and 4, and 11 videos (9.9%) were rated between 4 and 5. Fig 2 presents box plots of the mDISCERN, JAMA, and GQS score results for each language.

### Presenter gender analysis

Among the English-language videos, 14 videos were excluded because of unidentified presenter gender, 3 were animated, and 14 featured both male and female presenters. These 31 videos were excluded from the gender-based analysis, leaving 93 videos for comparison. After Bonferroni correction for multiple comparisons, the Kruskal‒Wallis test revealed no statistically significant differences in mDISCERN scores (adjusted *P* = 1.000), JAMA scores (adjusted *P* = 1.000), or GQS scores (adjusted *P* = 1.000) between male and female videos. Similarly, no significant differences were observed in the following quality metrics: liking (adjusted *P* = 1.000), disliking (adjusted *P* = 1.000), like ratio (adjusted *P* = 1.000), view ratio (adjusted *P* = 1.000), and VPI (adjusted *P* = 1.000).

Among the Spanish-language videos, 3 videos with unidentified presenter genders and 11 featuring both male and female presenters were excluded, resulting in 97 videos for analysis. However, no statistically significant differences in mDISCERN scores (adjusted *P* = 1.000), JAMA scores (adjusted *P* = 1.000), or GQS scores (adjusted *P* = 1.000) were found between sexes. The quality metrics also showed no significant differences: liking (adjusted *P* = 1.000), disliking (adjusted *P* = 1.000), like ratio (adjusted *P* = 1.000), view ratio (adjusted *P* = 1.000), and VPI (adjusted *P* = 1.000).

### Source group analysis

Universities and/or professional organisations produced 8 English videos, medical or profit-oriented companies 113, and independent users 3. Videos from independent users were excluded from the analysis. Statistically significant differences were observed between the groups for the mDISCERN (*P* < 0.001), JAMA (*P* < 0.001), and GQS (*P* < 0.001) scores. However, no significant differences were found for qualitative metrics, including likes (*P* = 0.764), dislikes (*P* = 0.936), like ratios (*P* = 0.408), view ratios (*P* = 0.410), and VPIs (*P* = 0.377). After Bonferroni correction for multiple comparisons, significant differences remained for mDISCERN (adjusted P < 0.008), JAMA (adjusted P < 0.008), and GQS (adjusted P < 0.008). No significant differences were observed for likes (adjusted P = 1.000), dislikes (adjusted P = 1.000), like ratio (adjusted P = 1.000), view ratio (adjusted P = 1.000), or VPI (adjusted P = 1.000).

Universities and/or professional organisations produced 10 Spanish-language videos, medical or profit-oriented companies produced 101 videos, and independent users produced 3 videos. As with the English-language videos, those produced by independent users were excluded from the analysis. After Bonferroni correction for multiple comparisons, no statistically significant differences were observed in mDISCERN (adjusted P = 0.488), JAMA (adjusted P = 0.256), or GQS scores (adjusted P = 0.352) between the groups. Likewise, no significant differences were found for any of the video quality metrics, including liking (adjusted P = 1.000), disliking (adjusted P = 1.000), like ratio (adjusted P = 1.000), view ratio (adjusted P = 1.000), and VPI (adjusted P = 1.000).

### Keyword search analysis between languages

An analysis was conducted to evaluate the potential differences in video visibility, measured via the VPI, across the four selected keywords.

No statistically significant differences in VPI were observed among the four topics in the English videos (P = 0.942). In contrast, a significant difference was found in the Spanish videos (P = 0.009). Post hoc analysis (Dunn’s correction) revealed that videos on “Atropina en gotas para control de miopía” had a significantly higher VPI than those on “lentes de contacto para control de miopía” (P = 0.004).

## Discussion

The growing public interest in myopia control reflects its status as a major global health issue, with the prevalence rising sharply, especially among children and adolescents. This trend has led to extensive research, and scientific evidence supports the effectiveness of interventions such as the use of specialised contact lenses, spectacle lenses, and low-dose atropine in slowing myopia progression and reducing ocular complications [30,31].

Despite the robustness of the scientific literature, the general public often seeks health information from online sources rather than from peer-reviewed articles. This highlights the importance of critically evaluating the accuracy and credibility of the content available on such platforms. While other social media networks, such as TikTok, Instagram, and Facebook, also play a role in health information dissemination, YouTube remains the most popular and widely used for longer educational content [19,21]. Recent umbrella reviews have highlighted that YouTube, along with X (formerly Twitter), Facebook, and Instagram, are the social networks most frequently used for health education, not only by the general public but also by healthcare professionals actively participating as content creators [32]. For this reason, and in line with previous studies, [9,11,12,19,26,27] we chose to focus our analysis on YouTube, recognising its relevance and potential impact on the public understanding of myopia control.

The findings of our study reveal significant variability in the quality and reliability of YouTube videos addressing myopia control in children, highlighting notable discrepancies between the information presented and the current scientific consensus. While some videos provided accurate, up-to-date, and evidence-based information consistent with current clinical guidelines, a substantial proportion fell short in terms of quality and scientific rigor. This raises important concerns regarding the potential spread of misinformation through widely used online platforms.

No significant differences were observed between languages for the JAMA or GQS scores. However, mDISCERN scores were significantly higher in Spanish-language videos than in English-language videos. This linguistic divergence is further reflected in our interobserver reliability analysis. English-language videos demonstrated moderate to good agreement across all evaluation tools, with ICC values of 0.768 for mDISCERN, 0.536 for JAMA, and 0.849 for GQS. Spanish-language videos showed good reliability for mDISCERN (ICC = 0.701) and GQS (ICC = 0.803), whereas JAMA demonstrated lower agreement (ICC = 0.319). These findings suggest that the consistency of ratings varied according to both the assessment instrument and language group. Notably, GQS achieved the highest reliability in both English- and Spanish-language videos, indicating that overall video quality may be evaluated more consistently than the specific criteria assessed by mDISCERN and JAMA. Other metrics, such as likes, dislikes, and like ratios, did not differ significantly between languages, suggesting that video popularity does not necessarily correlate with content quality. Interestingly, we observed higher view ratios and VPI scores in English-language videos, indicating greater visibility and reach, even when the scientific quality was not superior. This could be explained by the widespread use of English worldwide and the preference for many nonnative English speakers to search on the internet using this language.

Compared with those from medical or commercial companies or independent users, videos produced by universities or professional organisations tend to score higher on quality and reliability measures, especially in English. However, these high-quality videos represented a minority of the total sample, highlighting the gap between authoritative content and what is most accessible to the public. Gender analysis of the presenters revealed no significant effect on the perceived quality or popularity of the videos. Among the topics analysed, Spanish-language videos on atropine eye drops had a notably higher VPI, which indicates a particular public interest or engagement with this intervention in Spanish-speaking populations.

Several limitations must be acknowledged. First, our study was limited to videos in English and Spanish, potentially excluding relevant content in other widely spoken languages, such as Mandarin Chinese and Hindi [17]. Second, our analysis was restricted to YouTube, omitting other influential social media platforms where health information is frequently shared [33]. Importantly, adults and older individuals tend to seek health information primarily on platforms such as YouTube and Facebook, whereas younger audiences are increasingly turning to newer platforms such as TikTok and Instagram to satisfy their informational needs [28]. Moreover, as highlighted by Sufrate-Sorzano et al. (2024), healthcare professionals play a crucial role as active content creators in these digital environments, helping to bridge the gap between scientific evidence and public understanding and **combat** misinformation through the dissemination of clear and accessible information [32].

This generational difference in platform preference highlights the importance of expanding future research to include a broader range of social media channels to capture the diversity of information-seeking behaviours across age groups. Third, the dynamic nature of online content means that video availability, popularity, and engagement metrics can change rapidly, potentially affecting the reproducibility of our findings. Additionally, while we used validated tools to assess video quality and reliability, these instruments may not capture all dimensions of educational value or user engagement [34]. Finally, the exclusion of videos with disabled like/dislike or comment functions may have introduced selection bias, as these videos could differ systematically from those included.

Future research should continue to monitor the quality of online health information and explore interventions that encourage and facilitate the participation of healthcare professionals in digital education. Expanding this approach across multiple platforms and languages will be vital to ensure that accurate, up-to-date information reaches diverse audiences worldwide.

## Conclusions

While YouTube offers a vast repository of information on myopia control in children, the quality and reliability of the available content are highly variable. According to the validated scoring systems used, the platform is currently deficient in delivering comprehensive, evidence-based content on this topic. These deficiencies underscore the significant gap between the available scientific evidence and the information most readily accessible to the public [11]. An active role is needed by healthcare professionals and academic institutions for the creation and dissemination of reliable, evidence-based educational resources on popular digital platforms. By embracing e-professionalism and engaging directly with audiences through social media, health professionals can help ensure that online health information is accurate, trustworthy, and impactful, ultimately improving the quality of digital health education [32].

## Supporting information

S1 Table Search results, screening status, and performance highlights of collected YouTube videos on myopia control. (DOCX)

S1 File BASEDE_1. (XLSX)
