# Supplementary material for: Assessment of the consistency between YouTube videos and scientific evidence on paediatric myopia control
Source: PLoS One. 2026 Sep 23;21(9):e0357587. doi: 10.1371/journal.pone.0357587 (PMC13600497; doi:10.1371/journal.pone.0357587)
Supplement: Supplementary file 1 [file pone.0357587.s001.docx]

**Table S1**. Search results, screening status, and performance highlights of collected YouTube videos on myopia control.

A total of 400 YouTube videos and their corresponding web links were collected for this study. Of these, 235 videos that were actually analyzed have their links displayed in blue. The remaining 165 videos, marked in red, were excluded because they were duplicates, had disabled comments, likes, or dislikes, were in languages other than English or Spanish, or were not relevant to the topic of interest**. The 10 videos with the highest Video Power Index in the entire sample are highlighted in yellow. The 5 most viewed videos are highlighted in green. Additionally, the videos that achieved the best scores across all evaluation criteria, as well as the top five performing videos according to various measurement systems, are also indicated.**

- **Top ten VPI** (in order, yellow): 284,214,74,213,115,118,73,168,91,3
- **Top 5 most viewed** (in order, green): 284, 214,213, 74,115
- **Top Discern***:3,18,93,253,231,280,216,268,281,374,264,308,123,282,62,207,33
- **Top Jama*:** 18,268,33,12,93,216,308,282,246.
- **Top GQS***: 18,268,33,93,216,282,253,264,126,125,80,14,3.
- **Top 5 best scores** across all evaluation criteria: 18,93,216,268,282

***** All videos have the same punctuation

**Myopia control in children** (n=40)

| **Nº video** | **Link** |
| --- | --- |
| 1 | <https://www.youtube.com/watch?v=-z5M5Z-7Tpg&pp=ygUaTXlvcGlhIGNvbnRyb2wgaW4gY2hpbGRyZW4%3D> |
| 2 | <https://www.youtube.com/watch?v=6bfGYVrIWf4&pp=ygUaTXlvcGlhIGNvbnRyb2wgaW4gY2hpbGRyZW4%3D> |
| 3 | <https://www.youtube.com/watch?v=p-bC6krsZ2g&pp=ygUaTXlvcGlhIGNvbnRyb2wgaW4gY2hpbGRyZW4%3D> |
| 4 | <https://www.youtube.com/watch?v=XNzycjDLbTA&pp=ygUaTXlvcGlhIGNvbnRyb2wgaW4gY2hpbGRyZW4%3D> |
| 5 | <https://www.youtube.com/watch?v=IIPySV-ssB0&pp=ygUaTXlvcGlhIGNvbnRyb2wgaW4gY2hpbGRyZW4%3D> |
| 6 | <https://www.youtube.com/watch?v=WgVLVmwJX-s&pp=ygUaTXlvcGlhIGNvbnRyb2wgaW4gY2hpbGRyZW4%3D> |
| 7 | <https://www.youtube.com/watch?v=-pAZ2EXbafo&pp=ygUaTXlvcGlhIGNvbnRyb2wgaW4gY2hpbGRyZW4%3D> |
| 8 | <https://www.youtube.com/watch?v=79Wype5jMe0&pp=ygUaTXlvcGlhIGNvbnRyb2wgaW4gY2hpbGRyZW4%3D> |
| 9 | <https://www.youtube.com/watch?v=u_vqK1p5GB4&pp=ygUaTXlvcGlhIGNvbnRyb2wgaW4gY2hpbGRyZW4%3D> |
| 10 | <https://www.youtube.com/watch?v=ezP3oCRaBBQ&pp=ygUaTXlvcGlhIGNvbnRyb2wgaW4gY2hpbGRyZW4%3D> |
| 11 | <https://www.youtube.com/watch?v=seKmV-oXaFs&pp=ygUaTXlvcGlhIGNvbnRyb2wgaW4gY2hpbGRyZW4%3D> |
| 12 | <https://www.youtube.com/watch?v=DVnDVwQlDn4&pp=ygUaTXlvcGlhIGNvbnRyb2wgaW4gY2hpbGRyZW4%3D> |
| 13 | <https://www.youtube.com/watch?v=jgj3Ixp4zok&pp=ygUaTXlvcGlhIGNvbnRyb2wgaW4gY2hpbGRyZW4%3D> |
| 14 | <https://www.youtube.com/watch?v=Dz2UHcmyR4E&pp=ygUaTXlvcGlhIGNvbnRyb2wgaW4gY2hpbGRyZW4%3D> |
| 15 | <https://www.youtube.com/watch?v=R60TEJ0uGh0&pp=ygUaTXlvcGlhIGNvbnRyb2wgaW4gY2hpbGRyZW4%3D> |
| 16 | <https://www.youtube.com/watch?v=67ujpR3_CP8&pp=ygUaTXlvcGlhIGNvbnRyb2wgaW4gY2hpbGRyZW4%3D> |
| 17 | <https://www.youtube.com/watch?v=US3xuBmmWOc&pp=ygUaTXlvcGlhIGNvbnRyb2wgaW4gY2hpbGRyZW4%3D> |
| 18 | <https://www.youtube.com/watch?v=89nDb3GhpSQ&pp=ygUaTXlvcGlhIGNvbnRyb2wgaW4gY2hpbGRyZW4%3D> |
| 19 | <https://www.youtube.com/watch?v=ApSJnL2jqpA&pp=ygUaTXlvcGlhIGNvbnRyb2wgaW4gY2hpbGRyZW4%3D> |
| 20 | <https://www.youtube.com/watch?v=oSXgdq9QwdI&pp=ygUaTXlvcGlhIGNvbnRyb2wgaW4gY2hpbGRyZW4%3D> |
| 21 | <https://www.youtube.com/shorts/1minoCBxoBQ> |
| 22 | <https://www.youtube.com/watch?v=8kGA3HGukNc&pp=ygUaTXlvcGlhIGNvbnRyb2wgaW4gY2hpbGRyZW4%3D> |
| 23 | <https://www.youtube.com/watch?v=XNzycjDLbTA&pp=ygUaTXlvcGlhIGNvbnRyb2wgaW4gY2hpbGRyZW4%3D> |
| 24 | <https://www.youtube.com/watch?v=IIPySV-ssB0&pp=ygUaTXlvcGlhIGNvbnRyb2wgaW4gY2hpbGRyZW4%3D> |
| 25 | <https://www.youtube.com/watch?v=3dQG7ix4c5E&pp=ygUaTXlvcGlhIGNvbnRyb2wgaW4gY2hpbGRyZW4%3D> |
| 26 | <https://www.youtube.com/watch?v=Qqb7Mir7dLI&pp=ygUaTXlvcGlhIGNvbnRyb2wgaW4gY2hpbGRyZW4%3D> |
| 27 | <https://www.youtube.com/watch?v=_jn4mGmOa5A&pp=ygUaTXlvcGlhIGNvbnRyb2wgaW4gY2hpbGRyZW4%3D> |
| 28 | <https://www.youtube.com/watch?v=FOxvkcb9IRM&pp=ygUaTXlvcGlhIGNvbnRyb2wgaW4gY2hpbGRyZW4%3D> |
| 29 | <https://www.youtube.com/watch?v=pOZnNUK8FyE&pp=ygUaTXlvcGlhIGNvbnRyb2wgaW4gY2hpbGRyZW4%3D> |
| 30 | <https://www.youtube.com/watch?v=gdXoLRG1Zu0&pp=ygUaTXlvcGlhIGNvbnRyb2wgaW4gY2hpbGRyZW4%3D> |
| 31 | <https://www.youtube.com/watch?v=n-ZsjBrvQ-g&pp=ygUaTXlvcGlhIGNvbnRyb2wgaW4gY2hpbGRyZW4%3D> |
| 32 | <https://www.youtube.com/watch?v=DquUopd5C-4&pp=ygUaTXlvcGlhIGNvbnRyb2wgaW4gY2hpbGRyZW4%3D> |
| 33 | <https://www.youtube.com/watch?v=m586Vdogrsg&pp=ygUaTXlvcGlhIGNvbnRyb2wgaW4gY2hpbGRyZW4%3D> |
| 34 | <https://www.youtube.com/watch?v=WgVLVmwJX-s&pp=ygUaTXlvcGlhIGNvbnRyb2wgaW4gY2hpbGRyZW4%3D> |
| 35 | <https://www.youtube.com/watch?v=zy_-cDlG1zk&pp=ygUaTXlvcGlhIGNvbnRyb2wgaW4gY2hpbGRyZW4%3D> |
| 36 | <https://www.youtube.com/watch?v=SXo3RuYliEY&pp=ygUaTXlvcGlhIGNvbnRyb2wgaW4gY2hpbGRyZW4%3D> |
| 37 | <https://www.youtube.com/watch?v=mA1u_47fcEE&pp=ygUaTXlvcGlhIGNvbnRyb2wgaW4gY2hpbGRyZW4%3D> |
| 38 | <https://www.youtube.com/watch?v=wBQJcVLaJZM&pp=ygUaTXlvcGlhIGNvbnRyb2wgaW4gY2hpbGRyZW4%3D> |
| 39 | <https://www.youtube.com/watch?v=f3g6izhHuws&pp=ygUaTXlvcGlhIGNvbnRyb2wgaW4gY2hpbGRyZW4%3D> |
| 40 | <https://www.youtube.com/watch?v=DNdDYuOk0eE&pp=ygUaTXlvcGlhIGNvbnRyb2wgaW4gY2hpbGRyZW4%3D> |
| 41 | <https://www.youtube.com/watch?v=mDeRGtEoqLw&pp=ygUaTXlvcGlhIGNvbnRyb2wgaW4gY2hpbGRyZW4%3D> |
| 42 | <https://www.youtube.com/watch?v=wvvR6HnuaRI&pp=ygUaTXlvcGlhIGNvbnRyb2wgaW4gY2hpbGRyZW4%3D> |
| 43 | <https://www.youtube.com/shorts/xOwLJmO_7jo> |
| 44 | <https://www.youtube.com/watch?v=Gu2d-gegBz0&pp=ygUaTXlvcGlhIGNvbnRyb2wgaW4gY2hpbGRyZW4%3D> |
| 45 | <https://www.youtube.com/watch?v=Eg5HuUuSxkk&pp=ygUaTXlvcGlhIGNvbnRyb2wgaW4gY2hpbGRyZW4%3D> |
| 46 | <https://www.youtube.com/watch?v=HzAUOR0lsgw&pp=ygUaTXlvcGlhIGNvbnRyb2wgaW4gY2hpbGRyZW4%3D> |
| 47 | <https://www.youtube.com/watch?v=A28ePqKTkIg&pp=ygUaTXlvcGlhIGNvbnRyb2wgaW4gY2hpbGRyZW4%3D> |
| 48 | <https://www.youtube.com/watch?v=sbZfGTHn79E&pp=ygUaTXlvcGlhIGNvbnRyb2wgaW4gY2hpbGRyZW4%3D> |
| 49 | <https://www.youtube.com/watch?v=utpQ9SspZbo&pp=ygUaTXlvcGlhIGNvbnRyb2wgaW4gY2hpbGRyZW4%3D> |
| 50 | <https://www.youtube.com/watch?v=GCdZM4AlYXg&pp=ygUaTXlvcGlhIGNvbnRyb2wgaW4gY2hpbGRyZW4%3D> |

**Atropine eye drops for myopia** (n=32)

| **Nº video** | **Link** |
| --- | --- |
| 51 | <https://www.youtube.com/watch?v=6TtnKJSl-KE&pp=ygUdQXRyb3BpbmUgZXllIGRyb3BzIGZvciBteW9waWE%3D> |
| 52 | <https://www.youtube.com/watch?v=IfZyUC0preo&pp=ygUdQXRyb3BpbmUgZXllIGRyb3BzIGZvciBteW9waWE%3D> |
| 53 | <https://www.youtube.com/watch?v=OP-DCCT9d6I&pp=ygUdQXRyb3BpbmUgZXllIGRyb3BzIGZvciBteW9waWE%3D> |
| 54 | <https://www.youtube.com/watch?v=ZqpmliDGNvg&pp=ygUdQXRyb3BpbmUgZXllIGRyb3BzIGZvciBteW9waWE%3D> |
| 55 | <https://www.youtube.com/shorts/8gLSf9nrmTs> |
| 56 | <https://www.youtube.com/shorts/k8YMDCZpMPM> |
| 57 | <https://www.youtube.com/shorts/AOvBTw1MyRE> |
| 58 | <https://www.youtube.com/shorts/MFl4iOi5gdQ> |
| 59 | <https://www.youtube.com/shorts/Us2YHwUXJLM> |
| 60 | <https://www.youtube.com/watch?v=8kGA3HGukNc&pp=ygUdQXRyb3BpbmUgZXllIGRyb3BzIGZvciBteW9waWE%3D> |
| 61 | <https://www.youtube.com/watch?v=xQAgbhovmQY&pp=ygUdQXRyb3BpbmUgZXllIGRyb3BzIGZvciBteW9waWE%3D> |
| 62 | <https://www.youtube.com/watch?v=qJT_fBcPF64&pp=ygUdQXRyb3BpbmUgZXllIGRyb3BzIGZvciBteW9waWE%3D> |
| 63 | <https://www.youtube.com/watch?v=0oBb5GckC2E&pp=ygUdQXRyb3BpbmUgZXllIGRyb3BzIGZvciBteW9waWE%3D> |
| 64 | <https://www.youtube.com/watch?v=67ujpR3_CP8&pp=ygUdQXRyb3BpbmUgZXllIGRyb3BzIGZvciBteW9waWE%3D> |
| 65 | <https://www.youtube.com/watch?v=p-bC6krsZ2g&pp=ygUdQXRyb3BpbmUgZXllIGRyb3BzIGZvciBteW9waWE%3D> |
| 66 | <https://www.youtube.com/shorts/9gmhbv4-kpg> |
| 67 | <https://www.youtube.com/shorts/XssIpucDiSE> |
| 68 | <https://www.youtube.com/shorts/RR4q_Njs3Sg> |
| 69 | <https://www.youtube.com/shorts/vCjLxfr2XlQ> |
| 70 | <https://www.youtube.com/shorts/2YEvKuO6EY8> |
| 71 | <https://www.youtube.com/watch?v=YORPOV-o5aE&pp=ygUdQXRyb3BpbmUgZXllIGRyb3BzIGZvciBteW9waWE%3D> |
| 72 | <https://www.youtube.com/watch?v=gdXoLRG1Zu0&pp=ygUdQXRyb3BpbmUgZXllIGRyb3BzIGZvciBteW9waWE%3D> |
| 73 | <https://www.youtube.com/watch?v=7c3ZkTik95U&pp=ygUdQXRyb3BpbmUgZXllIGRyb3BzIGZvciBteW9waWE%3D> |
| 74 | <https://www.youtube.com/watch?v=RTZo5FmfpxE&pp=ygUdQXRyb3BpbmUgZXllIGRyb3BzIGZvciBteW9waWE%3D> |
| 75 | <https://www.youtube.com/watch?v=FbWuqVVdZIQ&pp=ygUdQXRyb3BpbmUgZXllIGRyb3BzIGZvciBteW9waWE%3D> |
| 76 | <https://www.youtube.com/watch?v=T19Z6GE95QA&pp=ygUdQXRyb3BpbmUgZXllIGRyb3BzIGZvciBteW9waWE%3D> |
| 77 | <https://www.youtube.com/watch?v=-MBu5aMi9co&pp=ygUdQXRyb3BpbmUgZXllIGRyb3BzIGZvciBteW9waWE%3D> |
| 78 | <https://www.youtube.com/watch?v=nE_JLsvEEvQ&pp=ygUdQXRyb3BpbmUgZXllIGRyb3BzIGZvciBteW9waWE%3D> |
| 79 | <https://www.youtube.com/watch?v=uxl_x-yqzps&pp=ygUdQXRyb3BpbmUgZXllIGRyb3BzIGZvciBteW9waWE%3D> |
| 80 | <https://www.youtube.com/watch?v=QcGcv15HIZ0&pp=ygUdQXRyb3BpbmUgZXllIGRyb3BzIGZvciBteW9waWE%3D> |
| 81 | <https://www.youtube.com/watch?v=-z5M5Z-7Tpg&pp=ygUdQXRyb3BpbmUgZXllIGRyb3BzIGZvciBteW9waWE%3D> |
| 82 | <https://www.youtube.com/watch?v=mF76pb2I8mE&pp=ygUdQXRyb3BpbmUgZXllIGRyb3BzIGZvciBteW9waWE%3D> |
| 83 | <https://www.youtube.com/shorts/PaAqqlZs8-E> |
| 84 | <https://www.youtube.com/watch?v=SHxExJmsKJ0&pp=ygUdQXRyb3BpbmUgZXllIGRyb3BzIGZvciBteW9waWE%3D> |
| 85 | <https://www.youtube.com/watch?v=cCu7AyNd5Mg&pp=ygUdQXRyb3BpbmUgZXllIGRyb3BzIGZvciBteW9waWE%3D> |
| 86 | <https://www.youtube.com/watch?v=qJT_fBcPF64&pp=ygUdQXRyb3BpbmUgZXllIGRyb3BzIGZvciBteW9waWE%3D> |
| 87 | <https://www.youtube.com/shorts/AOvBTw1MyRE> |
| 88 | <https://www.youtube.com/shorts/PaAqqlZs8-E> |
| 89 | <https://www.youtube.com/shorts/iOySGm_kVOU> |
| 90 | <https://www.youtube.com/shorts/R7Z1jxxrM2g> |
| 91 | <https://www.youtube.com/shorts/Y4P0wrmyEBM> |
| 92 | <https://www.youtube.com/shorts/ruQGF15J4Zw> |
| 93 | <https://www.youtube.com/watch?v=z8SSO4dSIK0&pp=ygUdQXRyb3BpbmUgZXllIGRyb3BzIGZvciBteW9waWE%3D> |
| 94 | <https://www.youtube.com/watch?v=sFq3hSaa8X8&pp=ygUdQXRyb3BpbmUgZXllIGRyb3BzIGZvciBteW9waWE%3D> |
| 95 | <https://www.youtube.com/watch?v=hGTyjJT1ToQ&pp=ygUdQXRyb3BpbmUgZXllIGRyb3BzIGZvciBteW9waWE%3D> |
| 96 | <https://www.youtube.com/shorts/Tl92dW5E6QU> |
| 97 | <https://www.youtube.com/shorts/Dq6bITY1VT0> |
| 98 | <https://www.youtube.com/shorts/LuKRne-dgow> |
| 99 | <https://www.youtube.com/shorts/0-M_KLTQE9o> |
| 100 | <https://www.youtube.com/shorts/lYgdMLfOjMk> |

**Contact lenses for myopia control** (n=28)

| **Nº video** | **Link** |
| --- | --- |
| 101 | <https://www.youtube.com/watch?v=HzAUOR0lsgw&pp=ygUiQ29udGFjdCBsZW5zZXMgZm9yIG15b3BpYSBjb250cm9sIA%3D%3D> |
| 102 | <https://www.youtube.com/watch?v=p-skBMkmRT4&pp=ygUiQ29udGFjdCBsZW5zZXMgZm9yIG15b3BpYSBjb250cm9sIA%3D%3D> |
| 103 | <https://www.youtube.com/watch?v=vO8CaZVTusc&pp=ygUiQ29udGFjdCBsZW5zZXMgZm9yIG15b3BpYSBjb250cm9sIA%3D%3D> |
| 104 | <https://www.youtube.com/watch?v=wtlrBLJIyis&pp=ygUiQ29udGFjdCBsZW5zZXMgZm9yIG15b3BpYSBjb250cm9sIA%3D%3D> |
| 105 | <https://www.youtube.com/watch?v=vciSjD9oifI&pp=ygUiQ29udGFjdCBsZW5zZXMgZm9yIG15b3BpYSBjb250cm9sIA%3D%3D> |
| 106 | <https://www.youtube.com/watch?v=5CgJxW6iaqA&pp=ygUiQ29udGFjdCBsZW5zZXMgZm9yIG15b3BpYSBjb250cm9sIA%3D%3D> |
| 107 | <https://www.youtube.com/shorts/XuOhnbcNakE> |
| 108 | <https://www.youtube.com/shorts/aNhO3RcGnHs> |
| 109 | <https://www.youtube.com/shorts/NQUJBXbRxYM> |
| 110 | <https://www.youtube.com/shorts/bxUv4EaJqh4> |
| 111 | <https://www.youtube.com/shorts/8G60OLYgZn8> |
| 112 | <https://www.youtube.com/watch?v=DVnDVwQlDn4&pp=ygUiQ29udGFjdCBsZW5zZXMgZm9yIG15b3BpYSBjb250cm9sIA%3D%3D> |
| 113 | <https://www.youtube.com/watch?v=xHJLkacb77Y&pp=ygUiQ29udGFjdCBsZW5zZXMgZm9yIG15b3BpYSBjb250cm9sIA%3D%3D> |
| 114 | <https://www.youtube.com/shorts/hBJL0E7onCA> |
| 115 | <https://www.youtube.com/shorts/cpxMmWBBV00> |
| 116 | <https://www.youtube.com/shorts/tNVqsB9plHM> |
| 117 | <https://www.youtube.com/shorts/WfxyTlhiaJs> |
| 118 | <https://www.youtube.com/shorts/VfS_zX7-7kE> |
| 119 | <https://www.youtube.com/watch?v=WK4Q5BAW2eA&pp=ygUiQ29udGFjdCBsZW5zZXMgZm9yIG15b3BpYSBjb250cm9sIA%3D%3D> |
| 120 | <https://www.youtube.com/watch?v=p-bC6krsZ2g&t=188s&pp=ygUiQ29udGFjdCBsZW5zZXMgZm9yIG15b3BpYSBjb250cm9sIA%3D%3D> |
| 121 | <https://www.youtube.com/watch?v=pTk5kLmZ8ec&pp=ygUiQ29udGFjdCBsZW5zZXMgZm9yIG15b3BpYSBjb250cm9sIA%3D%3D> |
| 122 | <https://www.youtube.com/watch?v=wtFb6uKPieY&pp=ygUiQ29udGFjdCBsZW5zZXMgZm9yIG15b3BpYSBjb250cm9sIA%3D%3D> |
| 123 | <https://www.youtube.com/watch?v=EoepQ9moasQ&pp=ygUiQ29udGFjdCBsZW5zZXMgZm9yIG15b3BpYSBjb250cm9sIA%3D%3D> |
| 124 | <https://www.youtube.com/watch?v=C2wnXG2UGRQ&pp=ygUiQ29udGFjdCBsZW5zZXMgZm9yIG15b3BpYSBjb250cm9sIA%3D%3D> |
| 125 | <https://www.youtube.com/watch?v=18eAQnyypRM&pp=ygUiQ29udGFjdCBsZW5zZXMgZm9yIG15b3BpYSBjb250cm9sIA%3D%3D> |
| 126 | <https://www.youtube.com/watch?v=qvNZyss3SW8&pp=ygUiQ29udGFjdCBsZW5zZXMgZm9yIG15b3BpYSBjb250cm9sIA%3D%3D> |
| 127 | <https://www.youtube.com/watch?v=P1ir94dgl6Q&pp=ygUiQ29udGFjdCBsZW5zZXMgZm9yIG15b3BpYSBjb250cm9sIA%3D%3D> |
| 128 | <https://www.youtube.com/watch?v=lohl0Ak08rI&pp=ygUiQ29udGFjdCBsZW5zZXMgZm9yIG15b3BpYSBjb250cm9sIA%3D%3D> |
| 129 | <https://www.youtube.com/watch?v=UoAVYHno3GI&pp=ygUiQ29udGFjdCBsZW5zZXMgZm9yIG15b3BpYSBjb250cm9sIA%3D%3D> |
| 130 | <https://www.youtube.com/watch?v=bWvMl48rvr8&pp=ygUiQ29udGFjdCBsZW5zZXMgZm9yIG15b3BpYSBjb250cm9sIA%3D%3D> |
| 131 | <https://www.youtube.com/watch?v=QHuI9MXKl_M&pp=ygUiQ29udGFjdCBsZW5zZXMgZm9yIG15b3BpYSBjb250cm9sIA%3D%3D> |
| 132 | <https://www.youtube.com/watch?v=hr_8kc35s00&pp=ygUiQ29udGFjdCBsZW5zZXMgZm9yIG15b3BpYSBjb250cm9sIA%3D%3D> |
| 133 | <https://www.youtube.com/watch?v=FOxvkcb9IRM&pp=ygUiQ29udGFjdCBsZW5zZXMgZm9yIG15b3BpYSBjb250cm9sIA%3D%3D> |
| 134 | <https://www.youtube.com/watch?v=p-skBMkmRT4&pp=ygUiQ29udGFjdCBsZW5zZXMgZm9yIG15b3BpYSBjb250cm9sIA%3D%3D> |
| 135 | <https://www.youtube.com/shorts/6kskmGGsIN8> |
| 136 | <https://www.youtube.com/shorts/WfxyTlhiaJs> |
| 137 | <https://www.youtube.com/shorts/EHV6DT8sbtY> |
| 138 | <https://www.youtube.com/shorts/VfS_zX7-7kE> |
| 139 | <https://www.youtube.com/shorts/UTzBAZj7pKs> |
| 140 | <https://www.youtube.com/shorts/fl4rgDA7AHc> |
| 141 | <https://www.youtube.com/watch?v=6fvj1qWZM9g&pp=ygUiQ29udGFjdCBsZW5zZXMgZm9yIG15b3BpYSBjb250cm9sIA%3D%3D> |
| 142 | <https://www.youtube.com/watch?v=YRqj1F8wq_s&pp=ygUiQ29udGFjdCBsZW5zZXMgZm9yIG15b3BpYSBjb250cm9sIA%3D%3D> |
| 143 | <https://www.youtube.com/watch?v=93Qf-yE3bmQ&pp=ygUiQ29udGFjdCBsZW5zZXMgZm9yIG15b3BpYSBjb250cm9sIA%3D%3D> |
| 144 | <https://www.youtube.com/shorts/N76DLIKKask> |
| 145 | <https://www.youtube.com/shorts/DUu1sCQsbw4> |
| 146 | <https://www.youtube.com/shorts/l6EHuUtsr6E> |
| 147 | <https://www.youtube.com/shorts/db8U1KLG2qI> |
| 148 | <https://www.youtube.com/shorts/4H1rfTeBu-0> |
| 149 | <https://www.youtube.com/watch?v=udzBO3JK7Gs&pp=ygUiQ29udGFjdCBsZW5zZXMgZm9yIG15b3BpYSBjb250cm9sIA%3D%3D> |
| 150 | <https://www.youtube.com/watch?v=I-lu148bBoI&pp=ygUiQ29udGFjdCBsZW5zZXMgZm9yIG15b3BpYSBjb250cm9sIA%3D%3D> |

**Spectacle lenses for myopia control** (n=24)

| **Nº video** | **Link** |
| --- | --- |
| 151 | <https://www.youtube.com/watch?v=DVnDVwQlDn4&pp=ygUkU3BlY3RhY2xlIGxlbnNlcyBmb3IgbXlvcGlhIGNvbnRyb2wg> |
| 152 | <https://www.youtube.com/watch?v=vO8CaZVTusc&pp=ygUkU3BlY3RhY2xlIGxlbnNlcyBmb3IgbXlvcGlhIGNvbnRyb2wg> |
| 153 | <https://www.youtube.com/watch?v=p-skBMkmRT4&pp=ygUkU3BlY3RhY2xlIGxlbnNlcyBmb3IgbXlvcGlhIGNvbnRyb2wg> |
| 154 | <https://www.youtube.com/watch?v=1w0k7aLwJgk&pp=ygUkU3BlY3RhY2xlIGxlbnNlcyBmb3IgbXlvcGlhIGNvbnRyb2wg> |
| 155 | <https://www.youtube.com/watch?v=18eAQnyypRM&pp=ygUkU3BlY3RhY2xlIGxlbnNlcyBmb3IgbXlvcGlhIGNvbnRyb2wg> |
| 156 | <https://www.youtube.com/watch?v=6fvj1qWZM9g&pp=ygUkU3BlY3RhY2xlIGxlbnNlcyBmb3IgbXlvcGlhIGNvbnRyb2wg> |
| 157 | <https://www.youtube.com/shorts/aNhO3RcGnHs> |
| 158 | <https://www.youtube.com/shorts/LjM4r-NFlLg> |
| 159 | <https://www.youtube.com/shorts/XuOhnbcNakE> |
| 160 | <https://www.youtube.com/shorts/GofbFl3_cL8> |
| 161 | <https://www.youtube.com/shorts/bxUv4EaJqh4> |
| 162 | <https://www.youtube.com/watch?v=xHJLkacb77Y&pp=ygUkU3BlY3RhY2xlIGxlbnNlcyBmb3IgbXlvcGlhIGNvbnRyb2wg> |
| 163 | <https://www.youtube.com/watch?v=hr_8kc35s00&pp=ygUkU3BlY3RhY2xlIGxlbnNlcyBmb3IgbXlvcGlhIGNvbnRyb2wg> |
| 164 | <https://www.youtube.com/watch?v=HzAUOR0lsgw&pp=ygUkU3BlY3RhY2xlIGxlbnNlcyBmb3IgbXlvcGlhIGNvbnRyb2wg> |
| 165 | <https://www.youtube.com/shorts/NQUJBXbRxYM> |
| 166 | <https://www.youtube.com/shorts/93-nRKiZeY0> |
| 167 | <https://www.youtube.com/shorts/UUO8U5MaQwA> |
| 168 | <https://www.youtube.com/shorts/Y4P0wrmyEBM> |
| 169 | <https://www.youtube.com/shorts/fl4rgDA7AHc> |
| 170 | <https://www.youtube.com/watch?v=wtFb6uKPieY&pp=ygUkU3BlY3RhY2xlIGxlbnNlcyBmb3IgbXlvcGlhIGNvbnRyb2wg> |
| 171 | <https://www.youtube.com/watch?v=GKYhZz0_Q5A&pp=ygUkU3BlY3RhY2xlIGxlbnNlcyBmb3IgbXlvcGlhIGNvbnRyb2wg> |
| 172 | <https://www.youtube.com/watch?v=FOxvkcb9IRM&pp=ygUkU3BlY3RhY2xlIGxlbnNlcyBmb3IgbXlvcGlhIGNvbnRyb2wg> |
| 173 | <https://www.youtube.com/watch?v=NaEaYo0dl6I&pp=ygUkU3BlY3RhY2xlIGxlbnNlcyBmb3IgbXlvcGlhIGNvbnRyb2wg> |
| 174 | <https://www.youtube.com/watch?v=L6Z2V2KmwtM&pp=ygUkU3BlY3RhY2xlIGxlbnNlcyBmb3IgbXlvcGlhIGNvbnRyb2wg> |
| 175 | <https://www.youtube.com/watch?v=WiZcItA8P9M&pp=ygUkU3BlY3RhY2xlIGxlbnNlcyBmb3IgbXlvcGlhIGNvbnRyb2wg> |
| 176 | <https://www.youtube.com/watch?v=HDt8lNIp0eQ&pp=ygUkU3BlY3RhY2xlIGxlbnNlcyBmb3IgbXlvcGlhIGNvbnRyb2wg> |
| 177 | <https://www.youtube.com/watch?v=qjkU7RT29_8&pp=ygUkU3BlY3RhY2xlIGxlbnNlcyBmb3IgbXlvcGlhIGNvbnRyb2wg> |
| 178 | <https://www.youtube.com/watch?v=qvNZyss3SW8&pp=ygUkU3BlY3RhY2xlIGxlbnNlcyBmb3IgbXlvcGlhIGNvbnRyb2wg> |
| 179 | <https://www.youtube.com/watch?v=p-bC6krsZ2g&pp=ygUkU3BlY3RhY2xlIGxlbnNlcyBmb3IgbXlvcGlhIGNvbnRyb2wg> |
| 180 | <https://www.youtube.com/watch?v=HLWnbyYV4RA&pp=ygUkU3BlY3RhY2xlIGxlbnNlcyBmb3IgbXlvcGlhIGNvbnRyb2wg> |
| 181 | <https://www.youtube.com/watch?v=fE9XP5WJS68&pp=ygUkU3BlY3RhY2xlIGxlbnNlcyBmb3IgbXlvcGlhIGNvbnRyb2wg> |
| 182 | <https://www.youtube.com/watch?v=ePn4xB7Id7o&pp=ygUkU3BlY3RhY2xlIGxlbnNlcyBmb3IgbXlvcGlhIGNvbnRyb2wg> |
| 183 | <https://www.youtube.com/watch?v=yJANJwuNwXQ&pp=ygUkU3BlY3RhY2xlIGxlbnNlcyBmb3IgbXlvcGlhIGNvbnRyb2wg> |
| 184 | <https://www.youtube.com/watch?v=p-skBMkmRT4&pp=ygUkU3BlY3RhY2xlIGxlbnNlcyBmb3IgbXlvcGlhIGNvbnRyb2wg> |
| 185 | <https://www.youtube.com/shorts/LjM4r-NFlLg> |
| 186 | <https://www.youtube.com/shorts/fl4rgDA7AHc> |
| 187 | <https://www.youtube.com/shorts/fWZHTtHZHtg> |
| 188 | <https://www.youtube.com/shorts/Wy17oj4U49A> |
| 189 | <https://www.youtube.com/shorts/Mncxf6yIxIw> |
| 190 | <https://www.youtube.com/shorts/1tJRdFqrvVM> |
| 191 | <https://www.youtube.com/shorts/XuOhnbcNakE> |
| 192 | <https://www.youtube.com/shorts/GofbFl3_cL8> |
| 193 | <https://www.youtube.com/watch?v=Lg9P7HfABbY&pp=ygUkU3BlY3RhY2xlIGxlbnNlcyBmb3IgbXlvcGlhIGNvbnRyb2wg> |
| 194 | <https://www.youtube.com/shorts/1tJRdFqrvVM> |
| 195 | <https://www.youtube.com/shorts/6ACB_eFQmz4> |
| 196 | <https://www.youtube.com/shorts/VT5DvSMaQ3g> |
| 197 | <https://www.youtube.com/shorts/x82MlVVDnds> |
| 198 | <https://www.youtube.com/shorts/DUu1sCQsbw4> |
| 199 | <https://www.youtube.com/watch?v=v3NHGoRSWVc&pp=ygUkU3BlY3RhY2xlIGxlbnNlcyBmb3IgbXlvcGlhIGNvbnRyb2wg> |
| 200 | <https://www.youtube.com/watch?v=vVOeWjmCoiM&pp=ygUkU3BlY3RhY2xlIGxlbnNlcyBmb3IgbXlvcGlhIGNvbnRyb2wg> |

**Control de miopía en niños** (n=40)

| **Nº video** | **Link** |
| --- | --- |
| 201 | <https://www.youtube.com/watch?v=X0s_sdammVI&pp=ygUbY29udHJvbCBkZSBtaW9waWEgZW4gbmnDsW9z> |
| 202 | <https://www.youtube.com/watch?v=CTV7BmeXarg&pp=ygUbY29udHJvbCBkZSBtaW9waWEgZW4gbmnDsW9z> |
| 203 | <https://www.youtube.com/watch?v=qIAnMPKnvAE&pp=ygUbY29udHJvbCBkZSBtaW9waWEgZW4gbmnDsW9z> |
| 204 | <https://www.youtube.com/watch?v=paxuZ3WmZiA&pp=ygUbY29udHJvbCBkZSBtaW9waWEgZW4gbmnDsW9z> |
| 205 | <https://www.youtube.com/watch?v=hnqgcLgTuF0&pp=ygUbY29udHJvbCBkZSBtaW9waWEgZW4gbmnDsW9z> |
| 206 | <https://www.youtube.com/watch?v=qHft1kdz3qM&pp=ygUbY29udHJvbCBkZSBtaW9waWEgZW4gbmnDsW9z> |
| 207 | <https://www.youtube.com/watch?v=nla6wUXxcww&pp=ygUbY29udHJvbCBkZSBtaW9waWEgZW4gbmnDsW9z> |
| 208 | <https://www.youtube.com/watch?v=ttUurKQ7Qi4&pp=ygUbY29udHJvbCBkZSBtaW9waWEgZW4gbmnDsW9z> |
| 209 | <https://www.youtube.com/watch?v=2Iy9V41RWO0&pp=ygUbY29udHJvbCBkZSBtaW9waWEgZW4gbmnDsW9z> |
| 210 | <https://www.youtube.com/watch?v=6F6psEIC8y0&pp=ygUbY29udHJvbCBkZSBtaW9waWEgZW4gbmnDsW9z> |
| 211 | <https://www.youtube.com/watch?v=X3DAqbXXTiI&pp=ygUbY29udHJvbCBkZSBtaW9waWEgZW4gbmnDsW9z> |
| 212 | <https://www.youtube.com/watch?v=FzcMgMOnEEw&pp=ygUbY29udHJvbCBkZSBtaW9waWEgZW4gbmnDsW9z> |
| 213 | <https://www.youtube.com/watch?v=1EatskI5Ds4&pp=ygUbY29udHJvbCBkZSBtaW9waWEgZW4gbmnDsW9z> |
| 214 | <https://www.youtube.com/watch?v=TtBOHvaw5pQ&pp=ygUbY29udHJvbCBkZSBtaW9waWEgZW4gbmnDsW9z> |
| 215 | <https://www.youtube.com/watch?v=BPMjEs6ILF4&pp=ygUbY29udHJvbCBkZSBtaW9waWEgZW4gbmnDsW9z> |
| 216 | <https://www.youtube.com/watch?v=ylv_JPvGRVw&pp=ygUbY29udHJvbCBkZSBtaW9waWEgZW4gbmnDsW9z> |
| 217 | <https://www.youtube.com/watch?v=7siZ5eH6RBk&pp=ygUbY29udHJvbCBkZSBtaW9waWEgZW4gbmnDsW9z> |
| 218 | <https://www.youtube.com/watch?v=QLG8mRMG4zM&pp=ygUbY29udHJvbCBkZSBtaW9waWEgZW4gbmnDsW9z> |
| 219 | <https://www.youtube.com/watch?v=Hqjh6QZ1WGM&pp=ygUbY29udHJvbCBkZSBtaW9waWEgZW4gbmnDsW9z> |
| 220 | <https://www.youtube.com/watch?v=PibM8RjFyPM&pp=ygUbY29udHJvbCBkZSBtaW9waWEgZW4gbmnDsW9z> |
| 221 | <https://www.youtube.com/watch?v=QQFQ4aV3ALw&pp=ygUbY29udHJvbCBkZSBtaW9waWEgZW4gbmnDsW9z> |
| 222 | <https://www.youtube.com/watch?v=KpTWMixUhYI&pp=ygUbY29udHJvbCBkZSBtaW9waWEgZW4gbmnDsW9z> |
| 223 | <https://www.youtube.com/watch?v=rXpw4bAZu_w&pp=ygUbY29udHJvbCBkZSBtaW9waWEgZW4gbmnDsW9z> |
| 224 | <https://www.youtube.com/watch?v=FsIurhvC1zA&pp=ygUbY29udHJvbCBkZSBtaW9waWEgZW4gbmnDsW9z> |
| 225 | <https://www.youtube.com/watch?v=pZ0jPk4fTiI&pp=ygUbY29udHJvbCBkZSBtaW9waWEgZW4gbmnDsW9z> |
| 226 | <https://www.youtube.com/watch?v=GHWJ7Jwn9qU&pp=ygUbY29udHJvbCBkZSBtaW9waWEgZW4gbmnDsW9z> |
| 227 | <https://www.youtube.com/watch?v=hnqgcLgTuF0&pp=ygUbY29udHJvbCBkZSBtaW9waWEgZW4gbmnDsW9z> |
| 228 | <https://www.youtube.com/watch?v=vSmk2Ws8wK0&pp=ygUbY29udHJvbCBkZSBtaW9waWEgZW4gbmnDsW9z> |
| 229 | <https://www.youtube.com/watch?v=abL9yqH3xYY&pp=ygUbY29udHJvbCBkZSBtaW9waWEgZW4gbmnDsW9z> |
| 230 | <https://www.youtube.com/watch?v=3VWeq5_zkZU&pp=ygUbY29udHJvbCBkZSBtaW9waWEgZW4gbmnDsW9z> |
| 231 | <https://www.youtube.com/watch?v=VnWLDPtvqyQ&pp=ygUbY29udHJvbCBkZSBtaW9waWEgZW4gbmnDsW9z> |
| 232 | <https://www.youtube.com/watch?v=qHft1kdz3qM&pp=ygUbY29udHJvbCBkZSBtaW9waWEgZW4gbmnDsW9z> |
| 233 | <https://www.youtube.com/watch?v=vtFw7OhPrGQ&pp=ygUbY29udHJvbCBkZSBtaW9waWEgZW4gbmnDsW9z> |
| 234 | <https://www.youtube.com/watch?v=FzcMgMOnEEw&pp=ygUbY29udHJvbCBkZSBtaW9waWEgZW4gbmnDsW9z> |
| 235 | <https://www.youtube.com/watch?v=KvkoZZ5xP80&t=479s&pp=ygUbY29udHJvbCBkZSBtaW9waWEgZW4gbmnDsW9z> |
| 236 | <https://www.youtube.com/watch?v=gVJXi3NL06M&pp=ygUbY29udHJvbCBkZSBtaW9waWEgZW4gbmnDsW9z> |
| 237 | <https://www.youtube.com/watch?v=BWAGlHSMX9I&pp=ygUbY29udHJvbCBkZSBtaW9waWEgZW4gbmnDsW9z> |
| 238 | <https://www.youtube.com/watch?v=Wqm9oXwOrs4&pp=ygUbY29udHJvbCBkZSBtaW9waWEgZW4gbmnDsW9z> |
| 239 | <https://www.youtube.com/watch?v=B-VKzCUN3xM&pp=ygUbY29udHJvbCBkZSBtaW9waWEgZW4gbmnDsW9z> |
| 240 | <https://www.youtube.com/watch?v=zzJYU4sk9cA&pp=ygUbY29udHJvbCBkZSBtaW9waWEgZW4gbmnDsW9z> |
| 241 | <https://www.youtube.com/watch?v=mVRjfENbj6E&pp=ygUbY29udHJvbCBkZSBtaW9waWEgZW4gbmnDsW9z> |
| 242 | <https://www.youtube.com/watch?v=ROwRRpHUNnY&pp=ygUbY29udHJvbCBkZSBtaW9waWEgZW4gbmnDsW9z> |
| 243 | <https://www.youtube.com/watch?v=XQSdQ-rp1UI&pp=ygUbY29udHJvbCBkZSBtaW9waWEgZW4gbmnDsW9z> |
| 244 | <https://www.youtube.com/watch?v=ySQYB3QBgrQ&pp=ygUbY29udHJvbCBkZSBtaW9waWEgZW4gbmnDsW9z> |
| 245 | <https://www.youtube.com/watch?v=nla6wUXxcww&pp=ygUbY29udHJvbCBkZSBtaW9waWEgZW4gbmnDsW9z> |
| 246 | <https://www.youtube.com/watch?v=vHbwxcKTXkE&pp=ygUbY29udHJvbCBkZSBtaW9waWEgZW4gbmnDsW9z> |
| 247 | <https://www.youtube.com/watch?v=KOBkZ9bYLJA&pp=ygUbY29udHJvbCBkZSBtaW9waWEgZW4gbmnDsW9z> |
| 248 | <https://www.youtube.com/watch?v=9UIjhUvZGbs&pp=ygUbY29udHJvbCBkZSBtaW9waWEgZW4gbmnDsW9z> |
| 249 | <https://www.youtube.com/watch?v=XqErpj1aM8w&pp=ygUbY29udHJvbCBkZSBtaW9waWEgZW4gbmnDsW9z> |
| 250 | <https://www.youtube.com/watch?v=5xPCQzti3o4&pp=ygUbY29udHJvbCBkZSBtaW9waWEgZW4gbmnDsW9z> |

**Atropina en gotas para control de miopía** (n=39)

| **Nº video** | **Link** |
| --- | --- |
| 251 | <https://www.youtube.com/watch?v=_BCmzS0joKs&pp=ygUpQXRyb3BpbmEgZW4gZ290YXMgcGFyYSBjb250cm9sIGRlIG1pb3DDrWE%3D> |
| 252 | <https://www.youtube.com/watch?v=iQ12k9_WYvk&pp=ygUpQXRyb3BpbmEgZW4gZ290YXMgcGFyYSBjb250cm9sIGRlIG1pb3DDrWE%3D> |
| 253 | <https://www.youtube.com/watch?v=VnWLDPtvqyQ&pp=ygUpQXRyb3BpbmEgZW4gZ290YXMgcGFyYSBjb250cm9sIGRlIG1pb3DDrWE%3D> |
| 254 | <https://www.youtube.com/watch?v=Xv-6xKtcDTg&pp=ygUpQXRyb3BpbmEgZW4gZ290YXMgcGFyYSBjb250cm9sIGRlIG1pb3DDrWE%3D> |
| 255 | <https://www.youtube.com/watch?v=dgfml6E1lmE&pp=ygUpQXRyb3BpbmEgZW4gZ290YXMgcGFyYSBjb250cm9sIGRlIG1pb3DDrWE%3D> |
| 256 | <https://www.youtube.com/watch?v=2OG-TQxl9EQ&pp=ygUpQXRyb3BpbmEgZW4gZ290YXMgcGFyYSBjb250cm9sIGRlIG1pb3DDrWE%3D> |
| 257 | <https://www.youtube.com/watch?v=q2ilKGkslmY&pp=ygUpQXRyb3BpbmEgZW4gZ290YXMgcGFyYSBjb250cm9sIGRlIG1pb3DDrWE%3D> |
| 258 | <https://www.youtube.com/watch?v=OHX4mjnlkTs&pp=ygUpQXRyb3BpbmEgZW4gZ290YXMgcGFyYSBjb250cm9sIGRlIG1pb3DDrWE%3D> |
| 259 | <https://www.youtube.com/watch?v=FX-JthXXwdQ&pp=ygUpQXRyb3BpbmEgZW4gZ290YXMgcGFyYSBjb250cm9sIGRlIG1pb3DDrWE%3D> |
| 260 | <https://www.youtube.com/watch?v=jZkfeC21Ye0&pp=ygUpQXRyb3BpbmEgZW4gZ290YXMgcGFyYSBjb250cm9sIGRlIG1pb3DDrWE%3D> |
| 261 | <https://www.youtube.com/shorts/L3bAnclY6Ak> |
| 262 | <https://www.youtube.com/watch?v=AOpR7hEGaKs&pp=ygUpQXRyb3BpbmEgZW4gZ290YXMgcGFyYSBjb250cm9sIGRlIG1pb3DDrWE%3D> |
| 263 | <https://www.youtube.com/shorts/pYigRqp_KNs> |
| 264 | <https://www.youtube.com/watch?v=Qsbr8XI_Ucc&pp=ygUpQXRyb3BpbmEgZW4gZ290YXMgcGFyYSBjb250cm9sIGRlIG1pb3DDrWE%3D> |
| 265 | <https://www.youtube.com/watch?v=wXuPFrDm76M&pp=ygUpQXRyb3BpbmEgZW4gZ290YXMgcGFyYSBjb250cm9sIGRlIG1pb3DDrWE%3D> |
| 266 | <https://www.youtube.com/watch?v=tQPZYMqeQC0&pp=ygUpQXRyb3BpbmEgZW4gZ290YXMgcGFyYSBjb250cm9sIGRlIG1pb3DDrWE%3D> |
| 267 | <https://www.youtube.com/shorts/tVla9gBXgVY> |
| 268 | <https://www.youtube.com/watch?v=6BFDPel2l2k&pp=ygUpQXRyb3BpbmEgZW4gZ290YXMgcGFyYSBjb250cm9sIGRlIG1pb3DDrWE%3D> |
| 269 | <https://www.youtube.com/watch?v=yTpq1R-osUU&pp=ygUpQXRyb3BpbmEgZW4gZ290YXMgcGFyYSBjb250cm9sIGRlIG1pb3DDrWE%3D> |
| 270 | <https://www.youtube.com/watch?v=SxA3173VPkQ&pp=ygUpQXRyb3BpbmEgZW4gZ290YXMgcGFyYSBjb250cm9sIGRlIG1pb3DDrWE%3D> |
| 271 | <https://www.youtube.com/watch?v=x0XV3w7XDUU&pp=ygUpQXRyb3BpbmEgZW4gZ290YXMgcGFyYSBjb250cm9sIGRlIG1pb3DDrWE%3D> |
| 272 | <https://www.youtube.com/watch?v=lq4FxOFkHIc&pp=ygUpQXRyb3BpbmEgZW4gZ290YXMgcGFyYSBjb250cm9sIGRlIG1pb3DDrWE%3D> |
| 273 | <https://www.youtube.com/watch?v=AXtPT-uEjD4&pp=ygUpQXRyb3BpbmEgZW4gZ290YXMgcGFyYSBjb250cm9sIGRlIG1pb3DDrWE%3D> |
| 274 | <https://www.youtube.com/shorts/RWQZKOqQBV0> |
| 275 | <https://www.youtube.com/watch?v=WnH6TeGdynY&pp=ygUpQXRyb3BpbmEgZW4gZ290YXMgcGFyYSBjb250cm9sIGRlIG1pb3DDrWE%3D> |
| 276 | <https://www.youtube.com/watch?v=X3DAqbXXTiI&pp=ygUpQXRyb3BpbmEgZW4gZ290YXMgcGFyYSBjb250cm9sIGRlIG1pb3DDrWE%3D> |
| 277 | <https://www.youtube.com/shorts/-i9b_poBHGY> |
| 278 | <https://www.youtube.com/watch?v=ySQYB3QBgrQ&pp=ygUpQXRyb3BpbmEgZW4gZ290YXMgcGFyYSBjb250cm9sIGRlIG1pb3DDrWE%3D> |
| 279 | <https://www.youtube.com/watch?v=tcIiGRb6wzg&pp=ygUpQXRyb3BpbmEgZW4gZ290YXMgcGFyYSBjb250cm9sIGRlIG1pb3DDrWE%3D> |
| 280 | <https://www.youtube.com/watch?v=ylv_JPvGRVw&pp=ygUpQXRyb3BpbmEgZW4gZ290YXMgcGFyYSBjb250cm9sIGRlIG1pb3DDrWE%3D> |
| 281 | <https://www.youtube.com/watch?v=aU7trlV_fc8&pp=ygUpQXRyb3BpbmEgZW4gZ290YXMgcGFyYSBjb250cm9sIGRlIG1pb3DDrWE%3D> |
| 282 | <https://www.youtube.com/watch?v=a-aJKEIwDOs&pp=ygUpQXRyb3BpbmEgZW4gZ290YXMgcGFyYSBjb250cm9sIGRlIG1pb3DDrWE%3D> |
| 283 | <https://www.youtube.com/watch?v=qIAnMPKnvAE&pp=ygUpQXRyb3BpbmEgZW4gZ290YXMgcGFyYSBjb250cm9sIGRlIG1pb3DDrWE%3D> |
| 284 | <https://www.youtube.com/watch?v=TtBOHvaw5pQ&pp=ygUpQXRyb3BpbmEgZW4gZ290YXMgcGFyYSBjb250cm9sIGRlIG1pb3DDrWE%3D> |
| 285 | <https://www.youtube.com/watch?v=X0s_sdammVI&t=136s&pp=ygUpQXRyb3BpbmEgZW4gZ290YXMgcGFyYSBjb250cm9sIGRlIG1pb3DDrWE%3D> |
| 286 | <https://www.youtube.com/watch?v=jlUQXZU66U4&pp=ygUpQXRyb3BpbmEgZW4gZ290YXMgcGFyYSBjb250cm9sIGRlIG1pb3DDrWE%3D> |
| 287 | <https://www.youtube.com/watch?v=CTV7BmeXarg&pp=ygUpQXRyb3BpbmEgZW4gZ290YXMgcGFyYSBjb250cm9sIGRlIG1pb3DDrWE%3D> |
| 288 | <https://www.youtube.com/shorts/MbIKq3sl9qY> |
| 289 | <https://www.youtube.com/watch?v=8KghuBdu53E&pp=ygUpQXRyb3BpbmEgZW4gZ290YXMgcGFyYSBjb250cm9sIGRlIG1pb3DDrWE%3D> |
| 290 | <https://www.youtube.com/watch?v=en8jBNDfNro&t=447s&pp=ygUpQXRyb3BpbmEgZW4gZ290YXMgcGFyYSBjb250cm9sIGRlIG1pb3DDrWE%3D> |
| 291 | <https://www.youtube.com/watch?v=bN0-fQpUPoE&pp=ygUpQXRyb3BpbmEgZW4gZ290YXMgcGFyYSBjb250cm9sIGRlIG1pb3DDrWE%3D> |
| 292 | <https://www.youtube.com/shorts/0q6eHUa8xZg> |
| 293 | <https://www.youtube.com/shorts/Oz5Gt7fmKTc> |
| 294 | <https://www.youtube.com/watch?v=y7zZcgHgiOA&pp=ygUpQXRyb3BpbmEgZW4gZ290YXMgcGFyYSBjb250cm9sIGRlIG1pb3DDrWE%3D> |
| 295 | <https://www.youtube.com/watch?v=6MIoddr-YvU&pp=ygUpQXRyb3BpbmEgZW4gZ290YXMgcGFyYSBjb250cm9sIGRlIG1pb3DDrWE%3D> |
| 296 | <https://www.youtube.com/watch?v=Tu5CRSDzVyE&pp=ygUpQXRyb3BpbmEgZW4gZ290YXMgcGFyYSBjb250cm9sIGRlIG1pb3DDrWE%3D> |
| 297 | <https://www.youtube.com/watch?v=cDc3OhO_hng&pp=ygUpQXRyb3BpbmEgZW4gZ290YXMgcGFyYSBjb250cm9sIGRlIG1pb3DDrWE%3D> |
| 298 | <https://www.youtube.com/watch?v=Aq6DGIBkHSM&pp=ygUpQXRyb3BpbmEgZW4gZ290YXMgcGFyYSBjb250cm9sIGRlIG1pb3DDrWE%3D> |
| 299 | <https://www.youtube.com/watch?v=QTK2VgdjsAY&pp=ygUpQXRyb3BpbmEgZW4gZ290YXMgcGFyYSBjb250cm9sIGRlIG1pb3DDrWE%3D> |
| 300 | <https://www.youtube.com/watch?v=iIOfz1ta0u8&pp=ygUpQXRyb3BpbmEgZW4gZ290YXMgcGFyYSBjb250cm9sIGRlIG1pb3DDrWE%3D> |

**Lentes de contacto para control de miopía** (n=24)

| **Nº video** | **Link** |
| --- | --- |
| 301 | <https://www.youtube.com/watch?v=KvkoZZ5xP80&t=76s&pp=ygUqTGVudGVzIGRlIGNvbnRhY3RvIHBhcmEgY29udHJvbCBkZSBtaW9ww61h> |
| 302 | <https://www.youtube.com/watch?v=9UIjhUvZGbs&pp=ygUqTGVudGVzIGRlIGNvbnRhY3RvIHBhcmEgY29udHJvbCBkZSBtaW9ww61h> |
| 303 | <https://www.youtube.com/watch?v=JKxn-ztFozE&pp=ygUqTGVudGVzIGRlIGNvbnRhY3RvIHBhcmEgY29udHJvbCBkZSBtaW9ww61h> |
| 304 | <https://www.youtube.com/watch?v=QQFQ4aV3ALw&pp=ygUqTGVudGVzIGRlIGNvbnRhY3RvIHBhcmEgY29udHJvbCBkZSBtaW9ww61h> |
| 305 | <https://www.youtube.com/shorts/v6kw6rS0DP4> |
| 306 | <https://www.youtube.com/watch?v=CiamQIlQal4&pp=ygUqTGVudGVzIGRlIGNvbnRhY3RvIHBhcmEgY29udHJvbCBkZSBtaW9ww61h> |
| 307 | <https://www.youtube.com/watch?v=74dPEMlvn2A&pp=ygUqTGVudGVzIGRlIGNvbnRhY3RvIHBhcmEgY29udHJvbCBkZSBtaW9ww61h> |
| 308 | <https://www.youtube.com/watch?v=wZ-dzDGHYok&pp=ygUqTGVudGVzIGRlIGNvbnRhY3RvIHBhcmEgY29udHJvbCBkZSBtaW9ww61h> |
| 309 | <https://www.youtube.com/watch?v=Wo6D1ATTRFg&pp=ygUqTGVudGVzIGRlIGNvbnRhY3RvIHBhcmEgY29udHJvbCBkZSBtaW9ww61h> |
| 310 | <https://www.youtube.com/watch?v=Td6kxUdoul8&pp=ygUqTGVudGVzIGRlIGNvbnRhY3RvIHBhcmEgY29udHJvbCBkZSBtaW9ww61h> |
| 311 | <https://www.youtube.com/watch?v=1EatskI5Ds4&pp=ygUqTGVudGVzIGRlIGNvbnRhY3RvIHBhcmEgY29udHJvbCBkZSBtaW9ww61h> |
| 312 | <https://www.youtube.com/watch?v=ORpXZN3rnGo&pp=ygUqTGVudGVzIGRlIGNvbnRhY3RvIHBhcmEgY29udHJvbCBkZSBtaW9ww61h> |
| 313 | <https://www.youtube.com/watch?v=NwVIdXSQ1Uw&pp=ygUqTGVudGVzIGRlIGNvbnRhY3RvIHBhcmEgY29udHJvbCBkZSBtaW9ww61h> |
| 314 | <https://www.youtube.com/watch?v=3POvTXQXyUc&pp=ygUqTGVudGVzIGRlIGNvbnRhY3RvIHBhcmEgY29udHJvbCBkZSBtaW9ww61h> |
| 315 | <https://www.youtube.com/watch?v=eGf4ZRUnz1U&pp=ygUqTGVudGVzIGRlIGNvbnRhY3RvIHBhcmEgY29udHJvbCBkZSBtaW9ww61h> |
| 316 | <https://www.youtube.com/watch?v=0gEwU5q9F44&pp=ygUqTGVudGVzIGRlIGNvbnRhY3RvIHBhcmEgY29udHJvbCBkZSBtaW9ww61h> |
| 317 | <https://www.youtube.com/watch?v=BtpFCa_mjOM&pp=ygUqTGVudGVzIGRlIGNvbnRhY3RvIHBhcmEgY29udHJvbCBkZSBtaW9ww61h> |
| 318 | <https://www.youtube.com/watch?v=G_JR-SgADMI&pp=ygUqTGVudGVzIGRlIGNvbnRhY3RvIHBhcmEgY29udHJvbCBkZSBtaW9ww61h> |
| 319 | <https://www.youtube.com/watch?v=KPxblfCfvGE&pp=ygUqTGVudGVzIGRlIGNvbnRhY3RvIHBhcmEgY29udHJvbCBkZSBtaW9ww61h> |
| 320 | <https://www.youtube.com/watch?v=fiItyVsx9R0&pp=ygUqTGVudGVzIGRlIGNvbnRhY3RvIHBhcmEgY29udHJvbCBkZSBtaW9ww61h> |
| 321 | <https://www.youtube.com/watch?v=en8jBNDfNro&pp=ygUqTGVudGVzIGRlIGNvbnRhY3RvIHBhcmEgY29udHJvbCBkZSBtaW9ww61h> |
| 322 | <https://www.youtube.com/watch?v=n9qizZNJeI4&pp=ygUqTGVudGVzIGRlIGNvbnRhY3RvIHBhcmEgY29udHJvbCBkZSBtaW9ww61h> |
| 323 | <https://www.youtube.com/watch?v=BPMjEs6ILF4&pp=ygUqTGVudGVzIGRlIGNvbnRhY3RvIHBhcmEgY29udHJvbCBkZSBtaW9ww61h> |
| 324 | <https://www.youtube.com/watch?v=_KL9yaUBJRg&pp=ygUqTGVudGVzIGRlIGNvbnRhY3RvIHBhcmEgY29udHJvbCBkZSBtaW9ww61h> |
| 325 | <https://www.youtube.com/watch?v=a2djROgkJXQ&pp=ygUqTGVudGVzIGRlIGNvbnRhY3RvIHBhcmEgY29udHJvbCBkZSBtaW9ww61h> |
| 326 | <https://www.youtube.com/watch?v=tOqC53VED9Q&pp=ygUqTGVudGVzIGRlIGNvbnRhY3RvIHBhcmEgY29udHJvbCBkZSBtaW9ww61h> |
| 327 | <https://www.youtube.com/watch?v=wuwAa4wm_V8&pp=ygUqTGVudGVzIGRlIGNvbnRhY3RvIHBhcmEgY29udHJvbCBkZSBtaW9ww61h> |
| 328 | <https://www.youtube.com/watch?v=YC_IP2JSOXM&pp=ygUqTGVudGVzIGRlIGNvbnRhY3RvIHBhcmEgY29udHJvbCBkZSBtaW9ww61h> |
| 329 | <https://www.youtube.com/shorts/U_vtlTLcIks> |
| 330 | <https://www.youtube.com/watch?v=gVJXi3NL06M&pp=ygUqTGVudGVzIGRlIGNvbnRhY3RvIHBhcmEgY29udHJvbCBkZSBtaW9ww61h> |
| 331 | <https://www.youtube.com/watch?v=cDc3OhO_hng&pp=ygUqTGVudGVzIGRlIGNvbnRhY3RvIHBhcmEgY29udHJvbCBkZSBtaW9ww61h> |
| 332 | <https://www.youtube.com/watch?v=qnLeYg-CLLc&pp=ygUqTGVudGVzIGRlIGNvbnRhY3RvIHBhcmEgY29udHJvbCBkZSBtaW9ww61h> |
| 333 | <https://www.youtube.com/watch?v=F01nMW4u18Y&pp=ygUqTGVudGVzIGRlIGNvbnRhY3RvIHBhcmEgY29udHJvbCBkZSBtaW9ww61h> |
| 334 | <https://www.youtube.com/shorts/0q6eHUa8xZg> |
| 335 | <https://www.youtube.com/watch?v=XQSdQ-rp1UI&pp=ygUqTGVudGVzIGRlIGNvbnRhY3RvIHBhcmEgY29udHJvbCBkZSBtaW9ww61h> |
| 336 | <https://www.youtube.com/watch?v=YFfnAchHis8&pp=ygUqTGVudGVzIGRlIGNvbnRhY3RvIHBhcmEgY29udHJvbCBkZSBtaW9ww61h> |
| 337 | <https://www.youtube.com/watch?v=tERcp8XZUPI&pp=ygUqTGVudGVzIGRlIGNvbnRhY3RvIHBhcmEgY29udHJvbCBkZSBtaW9ww61h> |
| 338 | <https://www.youtube.com/watch?v=RaRkWGiHJOk&pp=ygUqTGVudGVzIGRlIGNvbnRhY3RvIHBhcmEgY29udHJvbCBkZSBtaW9ww61h> |
| 339 | <https://www.youtube.com/watch?v=mtQFqa-TDAk&pp=ygUqTGVudGVzIGRlIGNvbnRhY3RvIHBhcmEgY29udHJvbCBkZSBtaW9ww61h> |
| 340 | <https://www.youtube.com/watch?v=ovr8MTK02Aw&pp=ygUqTGVudGVzIGRlIGNvbnRhY3RvIHBhcmEgY29udHJvbCBkZSBtaW9ww61h> |
| 341 | <https://www.youtube.com/watch?v=MxeB-i0FbG0&pp=ygUqTGVudGVzIGRlIGNvbnRhY3RvIHBhcmEgY29udHJvbCBkZSBtaW9ww61h> |
| 342 | <https://www.youtube.com/watch?v=fVuIJP1jv0c&pp=ygUqTGVudGVzIGRlIGNvbnRhY3RvIHBhcmEgY29udHJvbCBkZSBtaW9ww61h> |
| 343 | <https://www.youtube.com/watch?v=EAApUBNC_lc&pp=ygUqTGVudGVzIGRlIGNvbnRhY3RvIHBhcmEgY29udHJvbCBkZSBtaW9ww61h> |
| 344 | <https://www.youtube.com/watch?v=TtBOHvaw5pQ&pp=ygUqTGVudGVzIGRlIGNvbnRhY3RvIHBhcmEgY29udHJvbCBkZSBtaW9ww61h> |
| 345 | <https://www.youtube.com/watch?v=KHtfQQOWh24&pp=ygUqTGVudGVzIGRlIGNvbnRhY3RvIHBhcmEgY29udHJvbCBkZSBtaW9ww61h> |
| 346 | <https://www.youtube.com/watch?v=oba9ysuYKZw&pp=ygUqTGVudGVzIGRlIGNvbnRhY3RvIHBhcmEgY29udHJvbCBkZSBtaW9ww61h> |
| 347 | <https://www.youtube.com/shorts/U4CvEi5FyF4> |
| 348 | <https://www.youtube.com/watch?v=_1ZAW4OsR_8&pp=ygUqTGVudGVzIGRlIGNvbnRhY3RvIHBhcmEgY29udHJvbCBkZSBtaW9ww61h> |
| 349 | <https://www.youtube.com/watch?v=qchq7MdBg_I&pp=ygUqTGVudGVzIGRlIGNvbnRhY3RvIHBhcmEgY29udHJvbCBkZSBtaW9ww61h> |
| 350 | <https://www.youtube.com/watch?v=VQjaacsSF7E&pp=ygUqTGVudGVzIGRlIGNvbnRhY3RvIHBhcmEgY29udHJvbCBkZSBtaW9ww61h> |

**Gafas para control de miopía** (n=14)

| **Nº video** | **Link** |
| --- | --- |
| 351 | <https://www.youtube.com/watch?v=9UIjhUvZGbs&pp=ygUdR2FmYXMgcGFyYSBjb250cm9sIGRlIG1pb3DDrWE%3D> |
| 352 | <https://www.youtube.com/watch?v=KvkoZZ5xP80&t=76s&pp=ygUdR2FmYXMgcGFyYSBjb250cm9sIGRlIG1pb3DDrWE%3D> |
| 353 | <https://www.youtube.com/watch?v=wuwAa4wm_V8&pp=ygUdR2FmYXMgcGFyYSBjb250cm9sIGRlIG1pb3DDrWE%3D> |
| 354 | <https://www.youtube.com/watch?v=zzJYU4sk9cA&pp=ygUdR2FmYXMgcGFyYSBjb250cm9sIGRlIG1pb3DDrWE%3D> |
| 355 | <https://www.youtube.com/shorts/b5oJmFUXe6M> |
| 356 | <https://www.youtube.com/shorts/v6kw6rS0DP4> |
| 357 | <https://www.youtube.com/shorts/peu7_SxNkTA> |
| 358 | <https://www.youtube.com/shorts/3gRJzMfvTdg> |
| 359 | <https://www.youtube.com/shorts/iERS6qOGG4E> |
| 360 | <https://www.youtube.com/watch?v=QQFQ4aV3ALw&pp=ygUdR2FmYXMgcGFyYSBjb250cm9sIGRlIG1pb3DDrWE%3D> |
| 361 | <https://www.youtube.com/watch?v=3VWeq5_zkZU&pp=ygUdR2FmYXMgcGFyYSBjb250cm9sIGRlIG1pb3DDrWE%3D> |
| 362 | <https://www.youtube.com/watch?v=VZzmjyxvwB8&pp=ygUdR2FmYXMgcGFyYSBjb250cm9sIGRlIG1pb3DDrWE%3D> |
| 363 | <https://www.youtube.com/shorts/w7bpg1WyFzY> |
| 364 | <https://www.youtube.com/shorts/xbYl6Me8jMk> |
| 365 | <https://www.youtube.com/shorts/083SgKgsMyg> |
| 366 | <https://www.youtube.com/shorts/Ram5R2D0DEA> |
| 367 | <https://www.youtube.com/shorts/UuBBeU6uwF4> |
| 368 | <https://www.youtube.com/watch?v=74dPEMlvn2A&pp=ygUdR2FmYXMgcGFyYSBjb250cm9sIGRlIG1pb3DDrWE%3D> |
| 369 | <https://www.youtube.com/watch?v=cDc3OhO_hng&pp=ygUdR2FmYXMgcGFyYSBjb250cm9sIGRlIG1pb3DDrWE%3D> |
| 370 | <https://www.youtube.com/watch?v=en8jBNDfNro&pp=ygUdR2FmYXMgcGFyYSBjb250cm9sIGRlIG1pb3DDrWE%3D> |
| 371 | <https://www.youtube.com/watch?v=BPMjEs6ILF4&pp=ygUdR2FmYXMgcGFyYSBjb250cm9sIGRlIG1pb3DDrWE%3D> |
| 372 | <https://www.youtube.com/watch?v=fVuIJP1jv0c&pp=ygUdR2FmYXMgcGFyYSBjb250cm9sIGRlIG1pb3DDrWE%3D> |
| 373 | <https://www.youtube.com/watch?v=X3DAqbXXTiI&pp=ygUdR2FmYXMgcGFyYSBjb250cm9sIGRlIG1pb3DDrWE%3D> |
| 374 | <https://www.youtube.com/watch?v=a2djROgkJXQ&pp=ygUdR2FmYXMgcGFyYSBjb250cm9sIGRlIG1pb3DDrWE%3D> |
| 375 | <https://www.youtube.com/watch?v=jZkfeC21Ye0&pp=ygUdR2FmYXMgcGFyYSBjb250cm9sIGRlIG1pb3DDrWE%3D> |
| 376 | <https://www.youtube.com/watch?v=wZ-dzDGHYok&pp=ygUdR2FmYXMgcGFyYSBjb250cm9sIGRlIG1pb3DDrWE%3D> |
| 377 | <https://www.youtube.com/watch?v=w6iD05YnCxg&pp=ygUdR2FmYXMgcGFyYSBjb250cm9sIGRlIG1pb3DDrWE%3D> |
| 378 | <https://www.youtube.com/shorts/5mHl3ULK_3k> |
| 379 | <https://www.youtube.com/shorts/v6kw6rS0DP4> |
| 380 | <https://www.youtube.com/shorts/xbYl6Me8jMk> |
| 381 | <https://www.youtube.com/watch?v=XQSdQ-rp1UI&pp=ygUdR2FmYXMgcGFyYSBjb250cm9sIGRlIG1pb3DDrWE%3D> |
| 382 | <https://www.youtube.com/watch?v=TtBOHvaw5pQ&pp=ygUdR2FmYXMgcGFyYSBjb250cm9sIGRlIG1pb3DDrWE%3D> |
| 383 | <https://www.youtube.com/watch?v=tOqC53VED9Q&pp=ygUdR2FmYXMgcGFyYSBjb250cm9sIGRlIG1pb3DDrWE%3D> |
| 384 | <https://www.youtube.com/watch?v=HW6w_SFn_bA&pp=ygUdR2FmYXMgcGFyYSBjb250cm9sIGRlIG1pb3DDrWE%3D> |
| 385 | <https://www.youtube.com/shorts/0q6eHUa8xZg> |
| 386 | <https://www.youtube.com/watch?v=fiItyVsx9R0&pp=ygUdR2FmYXMgcGFyYSBjb250cm9sIGRlIG1pb3DDrWE%3D> |
| 387 | <https://www.youtube.com/watch?v=DoFtxi9VHj0&pp=ygUdR2FmYXMgcGFyYSBjb250cm9sIGRlIG1pb3DDrWE%3D> |
| 388 | <https://www.youtube.com/watch?v=_RE7Mx7VnwI&pp=ygUdR2FmYXMgcGFyYSBjb250cm9sIGRlIG1pb3DDrWE%3D> |
| 389 | <https://www.youtube.com/watch?v=BtpFCa_mjOM&pp=ygUdR2FmYXMgcGFyYSBjb250cm9sIGRlIG1pb3DDrWE%3D> |
| 390 | <https://www.youtube.com/shorts/uukK86flyX8> |
| 391 | <https://www.youtube.com/watch?v=1EatskI5Ds4&pp=ygUdR2FmYXMgcGFyYSBjb250cm9sIGRlIG1pb3DDrWE%3D> |
| 392 | <https://www.youtube.com/watch?v=ovr8MTK02Aw&pp=ygUdR2FmYXMgcGFyYSBjb250cm9sIGRlIG1pb3DDrWE%3D> |
| 393 | <https://www.youtube.com/watch?v=uiTbTaAfznQ&pp=ygUdR2FmYXMgcGFyYSBjb250cm9sIGRlIG1pb3DDrWE%3D> |
| 394 | <https://www.youtube.com/watch?v=KPxblfCfvGE&pp=ygUdR2FmYXMgcGFyYSBjb250cm9sIGRlIG1pb3DDrWE%3D> |
| 395 | <https://www.youtube.com/watch?v=G_JR-SgADMI&pp=ygUdR2FmYXMgcGFyYSBjb250cm9sIGRlIG1pb3DDrWE%3D> |
| 396 | <https://www.youtube.com/watch?v=gVJXi3NL06M&pp=ygUdR2FmYXMgcGFyYSBjb250cm9sIGRlIG1pb3DDrWE%3D> |
| 397 | <https://www.youtube.com/watch?v=eB-Nbz0oZL0&pp=ygUdR2FmYXMgcGFyYSBjb250cm9sIGRlIG1pb3DDrWE%3D> |
| 398 | <https://www.youtube.com/shorts/24wrvW2iT3g> |
| 399 | <https://www.youtube.com/watch?v=DPwR_H7kDRE&pp=ygUdR2FmYXMgcGFyYSBjb250cm9sIGRlIG1pb3DDrWE%3D> |
| 400 | <https://www.youtube.com/watch?v=Fpu1n7UTyAM&pp=ygUdR2FmYXMgcGFyYSBjb250cm9sIGRlIG1pb3DDrWE%3D> |
