# Supplementary material for: Assessment of the consistency between YouTube videos and scientific evidence on paediatric myopia control
Source: PLoS One. 2026 Sep 23;21(9):e0357587. doi: 10.1371/journal.pone.0357587 (PMC13600497; doi:10.1371/journal.pone.0357587)
Supplement: Supplementary file 2 [file pone.0357587.s004.docx]

**Point-by-point response to reviewers’**

Manuscript ID PONE-D-25-55052

Title: “Assessment of the consistency between YouTube videos and scientific evidence on paediatric myopia control”.

We would like to take this opportunity to express our gratitude to the reviewers for their very useful comments and suggestions, with which we believe our revised manuscript has been greatly improved.

●	All modifications in the revised manuscript have been highlighted using the

Track Changes tool for your convenience

●	Our responses to reviewers are in blue.

Please include the following items when submitting your revised manuscript:

- A letter that responds to each point raised by the academic editor and reviewer(s). You should upload this letter as a separate file labeled 'Response to Reviewers'.
- A marked-up copy of your manuscript that highlights changes made to the original version. You should upload this as a separate file labeled 'Revised Manuscript with Track Changes'.
- An unmarked version of your revised paper without tracked changes. You should upload this as a separate file labeled 'Manuscript'.

**RESPONSE TO THE EDITOR**

1. Please ensure that your manuscript meets PLOS ONE's style requirements, including those for file naming. The PLOS ONE style templates can be found at
<https://journals.plos.org/plosone/s/file?id=wjVg/PLOSOne_formatting_sample_main_body.pdf> and
[https://journals.plos.org/plosone/s/file?id=ba62/PLOSOne_formatting_sample_title_authors_affiliations.pdf](https://track.editorialmanager.com/CL0/https:%2F%2Fjournals.plos.org%2Fplosone%2Fs%2Ffile%3Fid=ba62%2FPLOSOne_formatting_sample_title_authors_affiliations.pdf/1/010f019f1bd99069-70909ca6-0c88-4b7d-bb1e-d494679dbfa8-000000/8ZByDT_8s0bX9cskfHnXRkdLfbn3ZFkXUGQ428GOMYQ=258)

We have carefully reviewed and adapted the entire manuscript to strictly comply with PLOS ONE’s style requirements, including section order and file naming conventions.

2. In your Methods section, please include additional information about your dataset and ensure that you have included a statement specifying whether the collection and analysis method complied with the terms and conditions for the source of the data.

Thank you for this crucial reminder. We have added a specific statement in the Methods section confirming compliance with YouTube's terms and conditions. Lines: 164-166

3. Please include captions for your Supporting Information files at the end of your manuscript, and update any in-text citations to match accordingly. Please see our Supporting Information guidelines for more information: <http://journals.plos.org/plosone/s/supporting-information>.

We have included the caption for Table S1 at the very end of the manuscript under a dedicated "Supporting Information" section. Lines: 657-659

4. If the reviewer comments include a recommendation to cite specific previously published works, please review and evaluate these publications to determine whether they are relevant and should be cited. There is no requirement to cite these works unless the editor has indicated otherwise.

No specific citations were requested by the editor or reviewers.

**RESPONSE TO THE REVIEWER’S**

**Reviewer 1**

This is very novel work for mostly discussed topic. Congratulations and best wishes!!
The methodology seems scientifically sound and the statistical analysis is also well done. The design and execution of the study is standard.

**Reviewer 2**

This manuscript attempts to answer questions about the quality of myopia educational videos on youtube. This is of interest in the community. The manuscript does a good job describing how videos were screened and selected for inclusion in the analysis, but there are significant concerns about the statistical approach to the data. Specifically, items of concern

Reviewer #2 raises substantive issues around the statistics. Specifically, there is the question of whether there was accounting for multiple comparisons. The other major issue is around the use of Cronbach's alpha, and whether this test is appropriate for these results. In addition, if Cronbach's alpha is the test of choice, please describe in detail how it should be interpreted. Please thoroughly address these comments and the other reviewer comments.

1. **Were adjustments made for multiple comparisons? It appears that multiple Mann-Whitney tests were performed and significance reported, but no mention of controlling for inflation of the Type I error with multiple comparisons.**

We agree with the Reviewer. To control for the inflation of Type I errors due to multiple comparisons, we have now explicitly applied the Bonferroni correction for all sub-analyses and post-hoc comparisons. Lines: 47; 255-257; 303-314: 326-328; 362-378; 387-391; 395-401.

2. **Application of Cronbach's alpha in the analysis as a measure of interobserver agreement.**

We appreciate this insightful methodological correction. The Reviewer is entirely correct that the Intraclass Correlation Coefficient (ICC) is the most appropriate metric for inter-rater reliability among the three optometrists. We have now computed and added the missing ICC values to Table 3. Lines: 293-297.

3. **Mention of the ICC in the methods, but no reported ICC values in the results and no discussion of ICC in results.These statistical questions reduce enthusiasm for the manuscript as written.**

We have included the ICC in the manuscript. Lines: 235-240 and 273-283

4. **Introduction: This introduction could be trimmed down. The first paragraph is a general discussion on prevalence and potential pathology. The second paragraph (lines 73-82) talks about different treatments, but this information that is widely known and not pertinent to the topic of the manuscript. I would delete the second paragraph to tighten up the manuscript.**

Following the Reviewer's excellent suggestion to tighten up the manuscript, we have deleted the original second paragraph concerning well-known clinical treatments (lines 88-97 in the original file). As a result, references 6 through 10, which were cited in this deleted passage, have been removed from the manuscript, and all subsequent references have been resequenced.

5. **Line 159: This is the first use of the VPI abbreviation in the manuscript proper. As such it should be spelled out and the abbreviation can be use thereafter.**

Fixed. The Video Power Index (VPI) is now spelled out in full upon its first occurrence in the text.

6. **Line 209: Not quite sure how this was double blind data. The 3 graders can be masked to each other’s scores, but there is no masking a patient in this study. You should clarify.**

We apologize for the confusion. The study was not double-blind in a clinical trial sense. The three evaluators were fully masked (blinded) to each other's scores and assignments to avoid cross-contamination. We have corrected the wording to "independent, masked evaluation". Lines: 227-229

7. **Lines 216-217: While it is good to look at normality of data, this is pertinent with a continuous variable. The scale values in these tools are ordinal data and not continuous, so a lack of normality is expected.**

We agree. Since assessment scales (mDISCERN, JAMA, GQS) yield ordinal data, non-normal distribution is inherently expected. We have rephrased this section to state that non-parametric tests were selected based on the ordinal nature of the data and confirmed via distribution analyses. Accordingly, we have rephrased the "Statistical analysis" section, line 233.

8. **Lines 223-224: I am not sure why you report in Table3 that you are looking at Cronbach’ alpha for interobserver reliability. Cronbach’s alpha looks at how well test items are measuring the same concept. This is typically used in survey development. Are you arguing that the 3 video quality metrics (mDISCERN, JAMA and GQS) are measuring the same concept, similar to different questions in a survey? While you compare the Cronbach alpha scores for the 3 devices, they are all asking something different about the video quality. For Interrater reliability for the video metric quality would be evaluated by the ICC, which you mention in the statistical methods but I do not see any mention of the ICC in the results or discussion.**

The Reviewer is entirely correct: Cronbach’s alpha reflects internal consistency among items rather than true inter-rater agreement for independent scoring tools, and the Intraclass Correlation Coefficient (ICC) is indeed the gold standard for this analysis. To address this, we have computed the ICC values instead of Cronbach’s aplha. Lines 249-252; 278-297.

9. **Lines 257-258: You are performing multiple Mann Whitney tests and report significance for the mDISCERN score. Did you adjust your p value to account for inflation of Type I errors?**

We acknowledge this important point raised by the Reviewer. To control for the inflation of Type I errors due to performing multiple Mann-Whitney U tests across different subgroups, we have now explicitly applied the Bonferroni correction for all pairwise and multiple sub-group comparisons.

Fortunately, the differences in the mDISCERN scores between the compared groups remain highly robust and statistically significant even after adjusting the P-values (P(adjusted) < 0.05). We have updated the P-values in the text and in Table 4 to reflect these adjusted values, and we have explicitly mentioned the use of the Bonferroni correction in the "Statistical analysis" section under Methods.

Lines 323-328

10. **Lines 263-265: Same comment on inflation of the Type I error. Did you adjust the p-value for multiple comparisons?**

We completely agree with the Reviewer. To maintain strict statistical rigor throughout the entire study and prevent Type I error inflation, the Bonferroni correction was systematically applied to this analysis as well.

11. **Lines 323-324 and Lines 331-332: Same question. Is this adjusted for multiple comparisons? The borderline significant values may not be significant when adjusting for multiple comparisons.**

Modified in the revised version

12. **Line 372: Is this significantly higher if accounting for multiple comparisons?**

Modified in the revised version. Lines: 450-461

13. **Line 390: “Several limitations…” should be a new paragraph**

Thank you for this formatting suggestion. We have modified the text accordingly.
