# Supplementary material for: Assessment of the consistency between YouTube videos and scientific evidence on paediatric myopia control
Source: PLoS One. 2026 Sep 23;21(9):e0357587. doi: 10.1371/journal.pone.0357587 (PMC13600497; doi:10.1371/journal.pone.0357587)
Supplement: Peer review history

# Peer review: Decision Letter 0

**DOI:** 10.1371/journal.pone.0357587.r001

**Authors:** Nick Fogt

1 Jul 2026

Dear Dr. Sunyer-Grau,

Thank you for submitting your manuscript to PLOS ONE. After careful consideration, we feel that it has merit but does not fully meet PLOS ONE’s publication criteria as it currently stands. Therefore, we invite you to submit a revised version of the manuscript that addresses the points raised during the review process.

Please submit your revised manuscript by Aug 15 2026 11:59PM. If you will need more time than this to complete your revisions, please reply to this message or contact the journal office at plosone@plos.org. When you're ready to submit your revision, log on to https://www.editorialmanager.com/pone/ and select the 'Submissions Needing Revision' folder to locate your manuscript file.

- A letter that responds to each point raised by the academic editor and reviewer(s). You should upload this letter as a separate file labeled 'Response to Reviewers'.
- A marked-up copy of your manuscript that highlights changes made to the original version. You should upload this as a separate file labeled 'Revised Manuscript with Track Changes'.
- An unmarked version of your revised paper without tracked changes. You should upload this as a separate file labeled 'Manuscript'.

If you would like to make changes to your financial disclosure, please include your updated statement in your cover letter. Guidelines for resubmitting your figure files are available below the reviewer comments at the end of this letter.

If applicable, we recommend that you deposit your laboratory protocols in protocols.io to enhance the reproducibility of your results. Protocols.io assigns your protocol its own identifier (DOI) so that it can be cited independently in the future. For instructions see: https://journals.plos.org/plosone/s/submission-guidelines#loc-laboratory-protocols. Additionally, PLOS ONE offers an option for publishing peer-reviewed Lab Protocol articles, which describe protocols hosted on protocols.io. Read more information on sharing protocols at https://plos.org/protocols?utm_medium=editorial-email&utm_source=authorletters&utm_campaign=protocols.

As the corresponding author, your ORCID iD is verified in the submission system and will appear in the published article. PLOS supports the use of ORCID, and we encourage all coauthors to register for an ORCID iD and use it as well. Please encourage your coauthors to verify their ORCID iD within the submission system before final acceptance, as unverified ORCID iDs will not appear in the published article. *Only*  the individual author can complete the verification step; PLOS staff *cannot*  verify ORCID iDs on behalf of authors.

We look forward to receiving your revised manuscript.

Kind regards,

Nick Fogt

Academic Editor

PLOS One

Journal Requirements:

When submitting your revision, we need you to address these additional requirements.

1. Please ensure that your manuscript meets PLOS ONE's style requirements, including those for file naming. The PLOS ONE style templates can be found at

https://journals.plos.org/plosone/s/file?id=wjVg/PLOSOne_formatting_sample_main_body.pdf and

https://journals.plos.org/plosone/s/file?id=ba62/PLOSOne_formatting_sample_title_authors_affiliations.pdf

2. In your Methods section, please include additional information about your dataset and ensure that you have included a statement specifying whether the collection and analysis method complied with the terms and conditions for the source of the data.

3. Please include captions for your Supporting Information files at the end of your manuscript, and update any in-text citations to match accordingly. Please see our Supporting Information guidelines for more information: http://journals.plos.org/plosone/s/supporting-information.

4. If the reviewer comments include a recommendation to cite specific previously published works, please review and evaluate these publications to determine whether they are relevant and should be cited. There is no requirement to cite these works unless the editor has indicated otherwise.

Additional Editor Comments:

Reviewer #2 raises substantive issues around the statistics. Specifically, there is the question of whether there was accounting for multiple comparisons. The other major issue is around the use of Cronbach's alpha, and whether this test is appropriate for these results. In addition, if Cronbach's alpha is the test of choice, please describe in detail how it should be interpreted. Please thoroughly address these comments and the other reviewer comments.

[Note: HTML markup is below. Please do not edit.]

Reviewers' comments:

Reviewer's Responses to Questions

**Comments to the Author**

1. Is the manuscript technically sound, and do the data support the conclusions?

Reviewer #1: Yes

Reviewer #2: Partly

**********

2. Has the statistical analysis been performed appropriately and rigorously?

Reviewer #1: Yes

Reviewer #2: No

**********

3. Have the authors made all data underlying the findings in their manuscript fully available?

The PLOS Data policy

Reviewer #1: Yes

Reviewer #2: Yes

**********

4. Is the manuscript presented in an intelligible fashion and written in standard English?

Reviewer #1: Yes

Reviewer #2: Yes

**********

Reviewer #1: This is very novel work for mostly discussed topic. Congratulations and best wishes!!

The methodology seems scientifically sound and the statistical analysis is also well done. The design and execution of the study is standard.

Reviewer #2: This manuscript attempts to answer questions about the quality of myopia educational videos on youtube. This is of interest in the community. The manuscript does a good job describing how videos were screened and selected for inclusion in the analysis, but there are significant concerns about the statistical approach to the data. Specifically, items of concern

1) Were adjustments made for multiple comparisons? It appears that multiple Mann-Whitney tests were performed and significance reported, but no mention of controlling for inflation of the Type I error with multiple comparisons.

2) Application of Cronbach's alpha in the analysis as a measure of interobserver agreement

3) Mention of the ICC in the methods, but no reported ICC values in the results and no discussion of ICC in results

These statistical questions reduce enthusiasm for the manuscript as written. Specific comments are below:

Introduction: This introduction could be trimmed down. The first paragraph is a general discussion on prevalence and potential pathology. The second paragraph (lines 73-82) talks about different treatments, but this information that is widely known and not pertinent to the topic of the manuscript. I would delete the second paragraph to tighten up the manuscript.

Line 159: This is the first use of the VPI abbreviation in the manuscript proper. As such it should be spelled out and the abbreviation can be use thereafter.

Line 209: Not quite sure how this was double blind data. The 3 graders can be masked to each other’s scores, but there is no masking a patient in this study. You should clarify.

Lines 216-217: While it is good to look at normality of data, this is pertinent with a continuous variable. The scale values in these tools are ordinal data and not continuous, so a lack of normality is expected.

Lines 223-224: I am not sure why you report in Figure 3 that you are looking at Cronbach’ alpha for interobserver reliability. Cronbach’s alpha looks at how well test items are measuring the same concept. This is typically used in survey development. Are you arguing that the 3 video quality metrics (mDISCERN, JAMA and GQS) are measuring the same concept, similar to different questions in a survey? While you compare the Cronbach alpha scores for the 3 devices, they are all asking something different about the video quality. For Interrater reliability for the video metric quality would be evaluated by the ICC, which you mention in the statistical methods but I do not see any mention of the ICC in the results or discussion.

Lines 257-258: You are performing multiple Mann Whitney tests and report significance for the mDISCERN score. Did you adjust your p value to account for inflation of Type I errors?

Lines 263-265: Same comment on inflation of the Type I error. Did you adjust the p-value for multiple comparisons?

Lines 323-324 and Lines 331-332: Same question. Is this adjusted for multiple comparisons? The borderline significant values may not be significant when adjusting for multiple comparisons.

Line 372: Is this significantly higher if accounting for multiple comparisons?

Line 390: “Several limitations…” should be a new paragraph

**********

what does this mean?). If published, this will include your full peer review and any attached files.

If you choose “no”, your identity will remain anonymous but your review may still be made public.

**Do you want your identity to be public for this peer review?**  For information about this choice, including consent withdrawal, please see our Privacy Policy

Reviewer #1: **Yes:** Raju Kaiti

Reviewer #2: No

**********

[NOTE: If reviewer comments were submitted as an attachment file, they will be attached to this email and accessible via the submission site. Please log into your account, locate the manuscript record, and check for the action link "View Attachments". If this link does not appear, there are no attachment files.]

To ensure your figures meet our technical requirements, please review our figure guidelines: https://journals.plos.org/plosone/s/figures

You may also use PLOS’s free figure tool, NAAS, to help you prepare publication quality figures: https://journals.plos.org/plosone/s/figures#loc-tools-for-figure-preparation.

NAAS will assess whether your figures meet our technical requirements by comparing each figure against our figure specifications.

# Author comment: Author response to Decision Letter 1

**DOI:** 10.1371/journal.pone.0357587.r002

28 Jul 2026

RESPONSE TO THE EDITOR

1. Please ensure that your manuscript meets PLOS ONE's style requirements, including those for file naming. The PLOS ONE style templates can be found at

https://journals.plos.org/plosone/s/file?id=wjVg/PLOSOne_formatting_sample_main_body.pdf and

https://journals.plos.org/plosone/s/file?id=ba62/PLOSOne_formatting_sample_title_authors_affiliations.pdf

We have carefully reviewed and adapted the entire manuscript to strictly comply with PLOS ONE’s style requirements, including section order and file naming conventions.

2. In your Methods section, please include additional information about your dataset and ensure that you have included a statement specifying whether the collection and analysis method complied with the terms and conditions for the source of the data.

Thank you for this crucial reminder. We have added a specific statement in the Methods section confirming compliance with YouTube's terms and conditions. Lines: 164-166

3. Please include captions for your Supporting Information files at the end of your manuscript, and update any in-text citations to match accordingly. Please see our Supporting Information guidelines for more information: http://journals.plos.org/plosone/s/supporting-information.

We have included the caption for Table S1 at the very end of the manuscript under a dedicated "Supporting Information" section. Lines: 657-659

4. If the reviewer comments include a recommendation to cite specific previously published works, please review and evaluate these publications to determine whether they are relevant and should be cited. There is no requirement to cite these works unless the editor has indicated otherwise.

No specific citations were requested by the editor or reviewers.

RESPONSE TO THE REVIEWER’S

Reviewer 1

This is very novel work for mostly discussed topic. Congratulations and best wishes!!

The methodology seems scientifically sound and the statistical analysis is also well done. The design and execution of the study is standard.

Reviewer 2

This manuscript attempts to answer questions about the quality of myopia educational videos on youtube. This is of interest in the community. The manuscript does a good job describing how videos were screened and selected for inclusion in the analysis, but there are significant concerns about the statistical approach to the data. Specifically, items of concern

Reviewer #2 raises substantive issues around the statistics. Specifically, there is the question of whether there was accounting for multiple comparisons. The other major issue is around the use of Cronbach's alpha, and whether this test is appropriate for these results. In addition, if Cronbach's alpha is the test of choice, please describe in detail how it should be interpreted. Please thoroughly address these comments and the other reviewer comments.

1. Were adjustments made for multiple comparisons? It appears that multiple Mann-Whitney tests were performed and significance reported, but no mention of controlling for inflation of the Type I error with multiple comparisons.

We agree with the Reviewer. To control for the inflation of Type I errors due to multiple comparisons, we have now explicitly applied the Bonferroni correction for all sub-analyses and post-hoc comparisons. Lines: 47; 255-257; 303-314: 326-328; 362-378; 387-391; 395-401.

2. Application of Cronbach's alpha in the analysis as a measure of interobserver agreement.

We appreciate this insightful methodological correction. The Reviewer is entirely correct that the Intraclass Correlation Coefficient (ICC) is the most appropriate metric for inter-rater reliability among the three optometrists. We have now computed and added the missing ICC values to Table 3. Lines: 293-297.

3. Mention of the ICC in the methods, but no reported ICC values in the results and no discussion of ICC in results.These statistical questions reduce enthusiasm for the manuscript as written.

We have included the ICC in the manuscript. Lines: 235-240 and 273-283

4. Introduction: This introduction could be trimmed down. The first paragraph is a general discussion on prevalence and potential pathology. The second paragraph (lines 73-82) talks about different treatments, but this information that is widely known and not pertinent to the topic of the manuscript. I would delete the second paragraph to tighten up the manuscript.

Following the Reviewer's excellent suggestion to tighten up the manuscript, we have deleted the original second paragraph concerning well-known clinical treatments (lines 88-97 in the original file). As a result, references 6 through 10, which were cited in this deleted passage, have been removed from the manuscript, and all subsequent references have been resequenced.

5. Line 159: This is the first use of the VPI abbreviation in the manuscript proper. As such it should be spelled out and the abbreviation can be use thereafter.

Fixed. The Video Power Index (VPI) is now spelled out in full upon its first occurrence in the text.

6. Line 209: Not quite sure how this was double blind data. The 3 graders can be masked to each other’s scores, but there is no masking a patient in this study. You should clarify.

We apologize for the confusion. The study was not double-blind in a clinical trial sense. The three evaluators were fully masked (blinded) to each other's scores and assignments to avoid cross-contamination. We have corrected the wording to "independent, masked evaluation". Lines: 227-229

7. Lines 216-217: While it is good to look at normality of data, this is pertinent with a continuous variable. The scale values in these tools are ordinal data and not continuous, so a lack of normality is expected.

We agree. Since assessment scales (mDISCERN, JAMA, GQS) yield ordinal data, non-normal distribution is inherently expected. We have rephrased this section to state that non-parametric tests were selected based on the ordinal nature of the data and confirmed via distribution analyses. Accordingly, we have rephrased the "Statistical analysis" section, line 233.

8. Lines 223-224: I am not sure why you report in Table3 that you are looking at Cronbach’ alpha for interobserver reliability. Cronbach’s alpha looks at how well test items are measuring the same concept. This is typically used in survey development. Are you arguing that the 3 video quality metrics (mDISCERN, JAMA and GQS) are measuring the same concept, similar to different questions in a survey? While you compare the Cronbach alpha scores for the 3 devices, they are all asking something different about the video quality. For Interrater reliability for the video metric quality would be evaluated by the ICC, which you mention in the statistical methods but I do not see any mention of the ICC in the results or discussion.

The Reviewer is entirely correct: Cronbach’s alpha reflects internal consistency among items rather than true inter-rater agreement for independent scoring tools, and the Intraclass Correlation Coefficient (ICC) is indeed the gold standard for this analysis. To address this, we have computed the ICC values instead of Cronbach’s aplha. Lines 249-252; 278-297.

9. Lines 257-258: You are performing multiple Mann Whitney tests and report significance for the mDISCERN score. Did you adjust your p value to account for inflation of Type I errors?

We acknowledge this important point raised by the Reviewer. To control for the inflation of Type I errors due to performing multiple Mann-Whitney U tests across different subgroups, we have now explicitly applied the Bonferroni correction for all pairwise and multiple sub-group comparisons.

Fortunately, the differences in the mDISCERN scores between the compared groups remain highly robust and statistically significant even after adjusting the P-values (P(adjusted) < 0.05). We have updated the P-values in the text and in Table 4 to reflect these adjusted values, and we have explicitly mentioned the use of the Bonferroni correction in the "Statistical analysis" section under Methods.

Lines 323-328

10. Lines 263-265: Same comment on inflation of the Type I error. Did you adjust the p-value for multiple comparisons?

We completely agree with the Reviewer. To maintain strict statistical rigor throughout the entire study and prevent Type I error inflation, the Bonferroni correction was systematically applied to this analysis as well.

11. Lines 323-324 and Lines 331-332: Same question. Is this adjusted for multiple comparisons? The borderline significant values may not be significant when adjusting for multiple comparisons.

Modified in the revised version

12. Line 372: Is this significantly higher if accounting for multiple comparisons?

Modified in the revised version. Lines: 450-461

13. Line 390: “Several limitations…” should be a new paragraph

Thank you for this formatting suggestion. We have modified the text accordingly.

Attachment Submitted filename: Response to Reviewers.docx

# Editor report: Decision Letter 1

**DOI:** 10.1371/journal.pone.0357587.r003

**Authors:** Nick Fogt

20 Aug 2026

<p>Assessment of the consistency between YouTube videos and scientific evidence on paediatric myopia control.

PONE-D-25-55052R1

Dear Dr. Sunyer-Grau,

We’re pleased to inform you that your manuscript has been judged scientifically suitable for publication and will be formally accepted for publication once it meets all outstanding technical requirements.

Within one week, you’ll receive an e-mail detailing the required amendments. When these have been addressed, you’ll receive a formal acceptance letter and your manuscript will be scheduled for publication.

An invoice will be generated when your article is formally accepted. Please note, if your institution has a publishing partnership with PLOS and your article meets the relevant criteria, all or part of your publication costs will be covered. Please make sure your user information is up-to-date by logging into Editorial Manager at Editorial Manager® and clicking the ‘Update My Information' link at the top of the page. For questions related to billing, please contact billing support.

If your institution or institutions have a press office, please notify them about your upcoming paper to help maximize its impact. If they’ll be preparing press materials, please inform our press team as soon as possible -- no later than 48 hours after receiving the formal acceptance. Your manuscript will remain under strict press embargo until 2 pm Eastern Time on the date of publication. For more information, please contact onepress@plos.org.

Kind regards,

Nick Fogt

Academic Editor

PLOS One

Additional Editor Comments (optional):

Thank you for addressing the reviewer comments. This paper is ready to move to the publication phase.

Reviewers' comments:

# Editor report: Acceptance letter

**DOI:** 10.1371/journal.pone.0357587.r004

**Authors:** Nick Fogt

PONE-D-25-55052R1

PLOS One

Dear Dr. Sunyer-Grau,

I'm pleased to inform you that your manuscript has been deemed suitable for publication in PLOS One. Congratulations! Your manuscript is now being handed over to our production team.

At this stage, our production department will prepare your paper for publication. This includes ensuring the following:

* All references, tables, and figures are properly cited

* All relevant supporting information is included in the manuscript submission,

* There are no issues that prevent the paper from being properly typeset

You will receive further instructions from the production team, including instructions on how to review your proof when it is ready. Please keep in mind that we are working through a large volume of accepted articles, so please give us a few days to review your paper and let you know the next and final steps.

Lastly, if your institution or institutions have a press office, please let them know about your upcoming paper now to help maximize its impact. If they'll be preparing press materials, please inform our press team within the next 48 hours. Your manuscript will remain under strict press embargo until 2 pm Eastern Time on the date of publication. For more information, please contact onepress@plos.org.

You will receive an invoice from PLOS for your publication fee after your manuscript has reached the completed accept phase. If you receive an email requesting payment before acceptance or for any other service, this may be a phishing scheme. Learn how to identify phishing emails and protect your accounts at https://explore.plos.org/phishing.

If we can help with anything else, please email us at customercare@plos.org.

Thank you for submitting your work to PLOS One and supporting open access.

Kind regards,

PLOS One Editorial Office Staff

on behalf of

Dr. Nick Fogt

Academic Editor

PLOS One
